# Rapid environmental effects on gut nematode susceptibility in rewilded mice

**DOI:** 10.1371/journal.pbio.2004108

**Published:** 2018-03-08

**Authors:** Jacqueline M. Leung, Sarah A. Budischak, Hao Chung The, Christina Hansen, Rowann Bowcutt, Rebecca Neill, Mitchell Shellman, P’ng Loke, Andrea L. Graham

**Affiliations:** 1 Department of Ecology and Evolutionary Biology, Princeton University, Princeton, New Jersey, United States of America; 2 Oxford University Clinical Research Unit, Wellcome Trust Major Overseas Programme, Vo Van Kiet, Ho Chi Minh City, Viet Nam; 3 Department of Microbiology, New York University School of Medicine, New York, New York, United States of America; Massachusetts Institute of Technology, United States of America

## Abstract

Genetic and environmental factors shape host susceptibility to infection, but how and how rapidly environmental variation might alter the susceptibility of mammalian genotypes remains unknown. Here, we investigate the impacts of seminatural environments upon the nematode susceptibility profiles of inbred C57BL/6 mice. We hypothesized that natural exposure to microbes might directly (e.g., via trophic interactions) or indirectly (e.g., via microbe-induced immune responses) alter the hatching, growth, and survival of nematodes in mice housed outdoors. We found that while C57BL/6 mice are resistant to high doses of nematode (*Trichuris muris*) eggs under clean laboratory conditions, exposure to outdoor environments significantly increased their susceptibility to infection, as evidenced by increased worm burdens and worm biomass. Indeed, mice kept outdoors harbored as many worms as signal transducer and activator of transcription 6 (STAT6) knockout mice, which are genetically deficient in the type 2 immune response essential for clearing nematodes. Using 16S ribosomal RNA sequencing of fecal samples, we discovered enhanced microbial diversity and specific bacterial taxa predictive of nematode burden in outdoor mice. We also observed decreased type 2 and increased type 1 immune responses in lamina propria and mesenteric lymph node (MLN) cells from infected mice residing outdoors. Importantly, in our experimental design, different groups of mice received nematode eggs either before or after moving outdoors. This contrasting timing of rewilding revealed that enhanced hatching of worms was not sufficient to explain the increased worm burdens; instead, microbial enhancement and type 1 immune facilitation of worm growth and survival, as hypothesized, were also necessary to explain our results. These findings demonstrate that environment can rapidly and significantly shape gut microbial communities and mucosal responses to nematode infections, leading to variation in parasite expulsion rates among genetically similar hosts.

## Author summary

The environment in which an individual resides is likely to change how she or he responds to infection. However, most of our understanding about host responses to infection arises from experimental studies conducted under uniform environmental conditions in the laboratory. We wished to investigate whether findings in the laboratory translate into the wild. Therefore, in this study, we placed common strains of laboratory mice into large, outdoor enclosures to investigate how a more natural environment might impact their ability to combat intestinal worm infections. We found that while mice are able to clear worm infections in the laboratory, mice residing outdoors harbored higher worm burdens and larger worms than their laboratory cousins. The longer the mice lived outdoors, the greater the number and size of worms in their guts. We found that outdoor mice harbored more diverse gut microbes and even specific bacteria that may have impacted worm growth and survival inside the mice. Mice kept outdoors also produced decreased immune responses of the type essential for worm expulsion. Together, these results demonstrate that the external environment significantly alters how a host responds to worms and germs in her or his gut, thereby leading to variation in the outcome of infections.

## Introduction

Individuals vary tremendously in their susceptibility to infection. For example, even with identical exposure rates, some hosts become heavily infected with parasitic worms, while others harbor none [1,2]. This variation in susceptibility impacts individual health and also shapes patterns of disease emergence [3], epidemiology [4], and control [5] at the population level. The causes of varied susceptibility are therefore important to understand yet can be complex to unravel. For example, host genetics explain some variation in susceptibility (e.g., [6,7]), but environmental heterogeneity in space and time (e.g., in abiotic variables such as ambient temperature [8] and biotic variables such as microbial diversity [9]) can also alter the susceptibility phenotype of a given genotype. As a result, the forces driving genetic susceptibility to infection in one environment may have no predictable effect, or even opposite effects, under other environmental conditions [10].

Unfortunately, despite the importance and ubiquity of variable environments and the demonstrated impact of environment upon human genetic susceptibility to disease [11], most experimental studies investigating mammalian susceptibility to infection are conducted under uniform environmental conditions in the laboratory. Such controlled laboratory conditions are no doubt critical to the discovery of molecular details of defense mechanisms. If susceptible genotypes described in the laboratory do not remain susceptible across all environments, however, or if the type, strength, or dynamics of the immune response are altered by the environment, it may become difficult to translate laboratory findings to the field. Here, we report a novel approach to bridging the divide between laboratory and field in mammalian immunology: rewilding experiments in which we quantify infection susceptibility, gut symbionts, and immune phenotypes of inbred strains of laboratory mice (*Mus musculus*) kept outdoors in seminatural conditions. We use the common C57BL/6 strain of *M*. *musculus* that has been a main focus of experimental immunology, placing individuals in outdoor environments that approximate the natural, farm-like habitats of agriculture-adapted human commensals such as *Mus* species. [12]. This experimental design allows us to control for factors such as host genetics, age, and sex in order to study the effects of environment on immune phenotype and susceptibility (and its converse, resistance) of hosts to infection.

Our rewilding approach builds upon other lab-to-wild bridging systems in three major ways: by bringing laboratory mice of selected genotypes into microbe-rich and otherwise complex environments, rather than bringing the microbes to them; by focusing on the gut as an arena of environmental exposure; and by investigating impacts of environment upon gut macrobiota (i.e., parasitic helminths) as well as microbiota. Indeed, recent studies have shown substantial immunophenotypic divergence between *M*. *musculus* laboratory strains versus field populations [13], in addition to significant effects of the microbial environment on how mice kept in the laboratory respond to infection [14,15]. For instance, even when laboratory mice are maintained continuously under hygienic conditions, differences in gut microbes associated with different commercial breeders can alter susceptibility to systemic infection (e.g., [16]). Importantly, mice raised under hygienic laboratory conditions lack the highly differentiated effector memory killer (CD8+) T cells found in wild and pet store–raised mice, which increases their susceptibility to viral and bacterial pathogens; “normalizing the environment” by housing laboratory-reared mice of the C57BL/6 strain with wild mice induces such T cells, improves resistance against pathogens, and thereby renders C57BL/6 mice a better immunological match for human adults [17]. This work demonstrates that the immune phenotype of a laboratory mouse genotype is altered when it is exposed to “dirty” [17] conspecifics. Laboratory mice reconstituted with the natural microbiota from wild mice also exhibit increased resistance against viral infections and mutagen- and inflammation-induced colorectal tumorigenesis [15]. While “clean” laboratory mice exhibited increased inflammatory cytokines, chemokines, and growth factors and, hence, collateral damage, during lethal influenza infection and tumorigenesis, those reconstituted with a more natural gut microbiota were better able to balance local and systemic inflammatory responses upon disease challenges, thus aiding in survival [15]. Furthermore, infection of laboratory mice with a series of common pathogenic viruses and parasites induces more natural immune phenotypes and alters host responses to vaccines [18].

Despite these advances, how external ecology impacts the internal ecology of the most prevalent symbionts of mammals remains a major knowledge gap. For instance, it is not known whether or how different environments, especially natural gut microbial exposures, impact the immune phenotype and gastrointestinal nematode susceptibility of mammalian genotypes. This gap is perhaps surprising, given that the gut presents a large surface area that is a primary interface with the external environment and given that billions of people harbor such ecosystems within their guts [19,20]. Interestingly, susceptibility to the nematode, *Heligmosomoides polygyrus*, is highly dependent on the mouse strain under laboratory cage conditions, but susceptibility differences disappear when the mice acquire infection at realistic, low transmission rates in an indoor arena [21,22]. These findings are consistent with the dose dependence of immune response induction by *H*. *polygyrus* [23]. However, no study to date has controlled both host genotype and parasite transmission to investigate impacts of a natural environment on host susceptibility to infection.

With our novel rewilding study design, we fill that gap. Our perspective is ultimately ecological, as we seek to understand how a mammal’s gastrointestinal interface with the external environment affects gut mucosal symbiont ecology and host immunology. Controlled laboratory studies (e.g., [15,17,18]) suggest that microbial exposure is the crucial aspect of nature that is missing from most of laboratory immunology, but the impact of microbes in an otherwise natural context remains unknown. Our experimental approach of putting mice on “farms” exposes them to environmental microbial diversity but also to other natural challenges, including the need to navigate a complex environment, find and build shelter, and endure variable weather conditions [24]. Importantly, we controlled for temperature and humidity differences between the laboratory and field so that we could rule out simple thermal preferences (which, for mice, is around 30°C [25], well above the usual “mouse house” temperature of 20–22°C) as a cause of any difference between susceptibility phenotypes in the laboratory versus field.

We expect a natural microbial environment to impact the nematode susceptibility of hosts via two main ecological processes: via direct (e.g., bottom-up) effects of altered microbes on hatching, growth, and development of worms or via top-down effects on the growth and survival of worms through changes to gut mucosal immune responses to infection. These two mechanisms can act independently or synergistically to shape host susceptibility to nematode infection. Guided by this community ecology logic and a detailed knowledge of the study system from laboratory experiments, our specific hypotheses are as follows. Our experiments pitted *M*. *musculus* genotypes in the laboratory and outdoors against *T*. *muris*, a natural colonic parasite of mice that is often used as a model system for *T*. *trichiura*, which infects over 450 million people [20]. The life cycle of *T*. *muris* follows a direct fecal–oral route. Ingested embryonated eggs travel to the cecum, where they hatch upon exposure to gut microbes [26,27] and initiate the release of infective larvae. We therefore hypothesized that any bottom-up, environmental effects on the composition and diversity of the gut microbiota could directly affect *T*. *muris* hatching, growth, and development and therefore host susceptibility. To investigate the effect of the gut microbiota on *T*. *muris* hatching per se, we manipulated the timing of rewilding (see below) so that different groups were infected either before or after exposure to environmental microbes.

Next, as *T*. *muris* larvae grow and molt, they move from the base of the crypts into the gut lumen and mature into adult worms. Along the way, the type of immune response mounted plays a critical role in determining host susceptibility to persistent infection. The generation of type 2 (Th2) cytokines, particularly interleukins (IL)-13 and IL-4, is associated with parasite expulsion through increased epithelial cell turnover, mucus production, and muscle hypercontractility [28]. The expression of these worm-clearing cytokines is promoted by a transcription factor, signal transducer and activator of transcription 6 (STAT6), and mice deficient in STAT6 (STAT6-/-) are highly susceptible to nematode infections [29]. Binding of IL-4 to the IL-4 receptor, IL-4R, activates STAT6 [30], and hence blocking IL-4 function in vivo has been shown to prevent *T*. *muris* expulsion [31]. *T*. *muris* burdens in STAT6-/- mice are therefore high and often used to maintain the *T*. *muris* life cycle [32]. Conversely, type 1 (Th1) cytokines, such as interferon-gamma (IFNγ) [33,34], pro-inflammatory cytokines such as IL-17 [35], and the regulatory cytokine, IL-10 [36], lead to increased susceptibility to high doses of *T*. *muris* and the establishment of chronic infections. Similarly, low infective doses of *T*. *muris* in laboratory mice favor the development of a susceptibility-associated Th1 response [37], whereas higher infective doses (>200 eggs) lead to the development of an effective Th2 response and hence resistance to *T*. *muris* in laboratory C57BL/6 mice [38]. We therefore further hypothesized that any environmental effects on the type of immune response mounted (e.g., top-down effects on high doses of worms) could alter laboratory-defined susceptibility profiles. We thus compared immune and infection profiles of rewilded C57BL/6 mice to susceptible STAT6-/- mice. Lastly, we hypothesized that gut microbes might have indirect effects on nematode susceptibility, e.g., that microbes altered induced immune responses and thus top-down control of worms.

**Fig 1:**
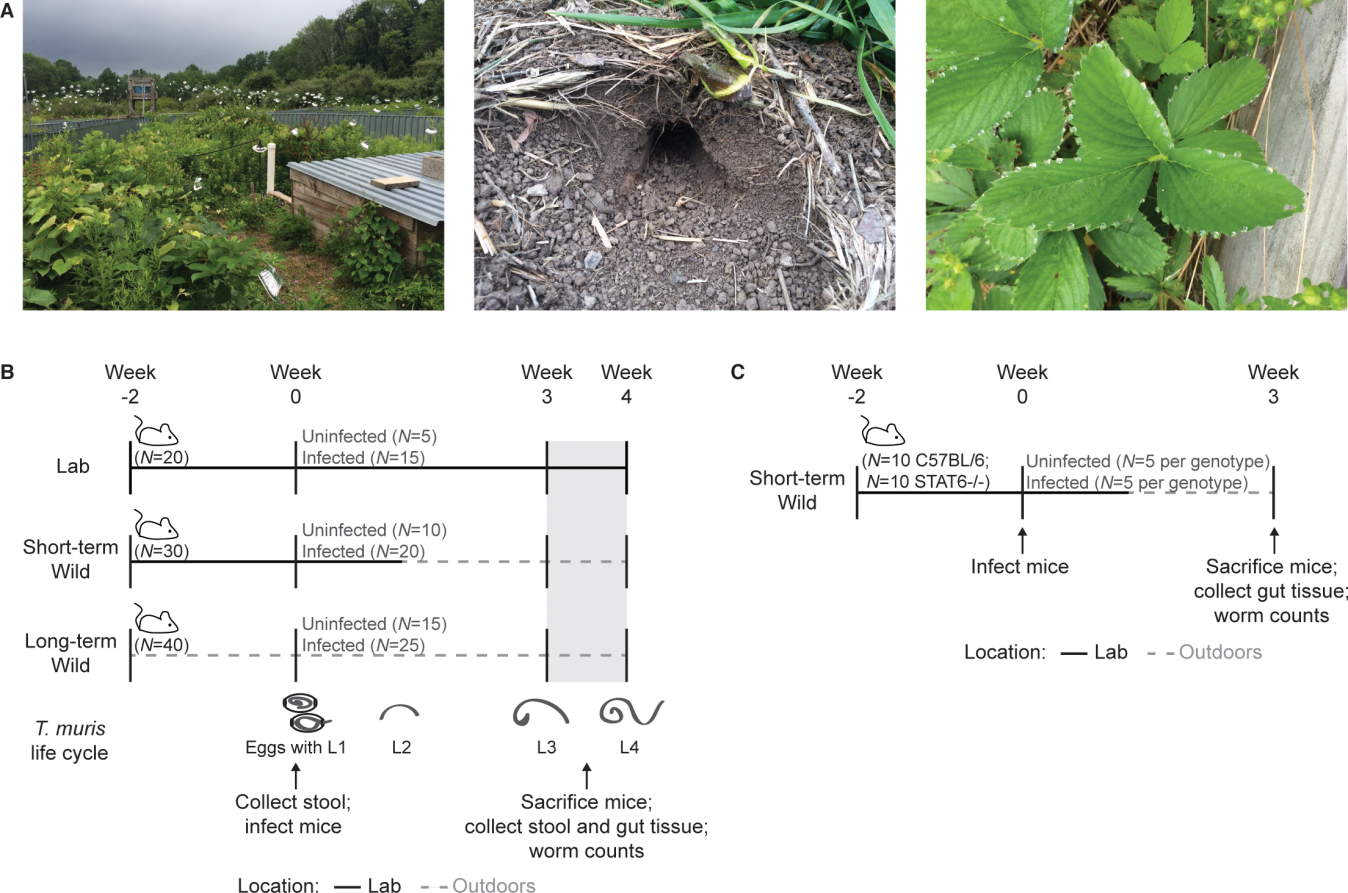
Experimental design. (**A**) Images of the outdoor enclosures depicting the environments that the Short-term Wild and Long-term Wild mice were exposed to. Images shown include a representative wedge of the enclosure, burrows created by C57BL/6 mice in the dirt, and natural sources of water for the mice. (**B**) Treatment groups and sampling time points for investigating the effects of environmental variation on *Trichuris muris* infection in C57BL/6 mice. The *T*. *muris* life cycle [40] is also depicted in relation to the study time line to illustrate the different nematode growth stages during the experiment. (**C**) Treatment groups and sampling time points for investigating the effects of environmental variation on *T*. *muris* infection in C57BL/6 and STAT6-/- mice. L1, larval stage 1; STAT6-/-, mouse deficient in STAT6.

A key innovation of our study is the rewilding of established strains of laboratory mice by introducing them into outdoor enclosures [39], which include natural soil, weather, and vegetation but also protection against predation (Fig 1A). Importantly, we varied the timing of the environmental shift relative to the timing of worm infection to explore how different worm stages were impacted by altered microbes and immunity, as follows (Fig 1B). The Lab mice group remained in the laboratory for the duration of the study. The Short-term Wild mice remained in the laboratory initially, were infected with *T*. *muris* while in the laboratory, and were then moved to the outdoor enclosures 10 days postinfection (p.i.). According to the life cycle of *T*. *muris*, this allowed for the hatching of *T*. *muris* eggs and the molting of larval stage 1 (L1) to larval stage 2 (L2) in the laboratory before the Short-term Wild mice moved outdoors (Fig 1B and 1C). Long-term Wild mice resided in the outdoor enclosures for the duration of the study, receiving the nematode inoculum after 2 weeks outdoors. Mice from all three treatment groups were sampled at two time points following infection (at 3 weeks and 4 weeks p.i.) to investigate the dynamics of susceptibility and immune variation (Fig 1B). With this experimental design, we aimed to determine whether *T*. *muris* hatching, growth rates, and subsequent survival were all impacted by the change in host environment, or whether an effect of the external environment on just one part of the nematode life cycle (e.g., hatching) was paramount.

## Results

### Increased worm survival and biomass in mice kept outdoors

**Fig 2:**
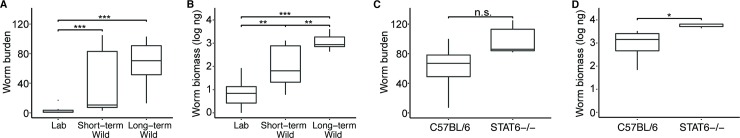
*Trichuris muris* worm burdens and worm biomass in C57BL/6 mice in laboratory versus outdoor environments at 3 weeks p.i. (**A**) Worm burdens and (**B**) worm biomass from infected C57BL/6 mice residing in laboratory and outdoor environments at 3 weeks p.i. Lab: *N* = 8; Short-term Wild: *N* = 10; Long-term Wild: *N* = 10. (**C**) Worm burdens and (**D**) worm biomass from C57BL/6 and STAT6-/- mice residing outdoors for the short-term at 3 weeks p.i. C57BL/6: *N* = 4; STAT6-/-: *N* = 5. Box centers show the medians, and the upper and lower box edges correspond to the 25th and 75th percentiles. Whiskers extend 1.5 times the interquartile range. Please see Materials and methods for further information on potential sources of variation and associated statistical tests. Asterisks denote significance as *P < 0.05, **P < 0.01, ***P < 0.001. https://doi.org/10.5061/dryad.h9g697r. ng, nanograms; n.s., not significant; p.i., postinfection; STAT6-/-, mouse deficient in STAT6.

Persistent worm burden and a large biomass of nematodes are common quantitative indices of host susceptibility [41], so we focus on those here. Worm counts obtained from the ceca revealed that Lab mice harbored few worms at 3 weeks p.i., whereas Short-term Wild and Long-term Wild mice had significantly higher worm burdens at this time point (Fig 2A; Long-term Wild versus Lab: *z* = 6.015; Short-term Wild versus Lab: *z* = 4.980; both *P* < 0.0001). The worm burdens of the two wild groups did not differ from each other (*P* > 0.4). Hosts residing outdoors also harbored larger worm biomasses at 3 weeks p.i., compared to laboratory mice (Fig 2B). Indeed, the mean worm biomass in Long-term Wild mice was significantly greater than in both Short-term Wild and Lab mice (Fig 2B; Long-term Wild versus Short-term Wild: *z* = 3.443 and *P* = 0.00165; Long-term Wild versus Lab: *z* = 6.354 and *P* < 0.0001). The Short-term Wild mice also harbored larger worm biomasses than Lab mice (Fig 2B; *z* = 3.372 and *P* = 0.00208). Unsurprisingly, given that worm length (along with width) is used to calculate biomass, significant differences in worm length across environments mirrored the worm biomass data: worms from Long-term Wild mice had the largest average helminth length, followed by worms from the Short-term Wild mice. Lab mice had the smallest average worm length (Long-term Wild versus Short-term Wild: *z* = 3.800 and *P* = 0.0004; Long-term Wild versus Lab: *z* = 5.719 and *P* < 0.0001; Short-term Wild versus Lab: *z* = 2.428 and *P* = 0.04). Furthermore, worm burdens of C57BL/6 mice residing outdoors for the short term were statistically indistinguishable from the burdens of genetically worm-susceptible STAT6-/- mice at 3 weeks p.i. (Fig 2C; *z* = 1.36 and *P* = 0.174), although STAT6-/- mice did harbor greater nematode biomass (Fig 2D; *z* = 2.51 and *P* = 0.0121) and average worm length (|*z*| = 5.622 and *P* < 0.0001). No statistical differences in worm burdens were observed between the Short-term Wild mice in the main experiment (Fig 2A) and the C57BL/6 mice in the STAT6-/- experiment (Fig 2C) (unpaired *t* test, *P* = 0.469), although the worm biomasses from the C57BL/6 mice in the STAT6-/- experiment were greater (unpaired *t* test, *P* = 0.03) (Fig 2B and 2D).

By 4 weeks p.i., infected C57BL/6 mice harbored few worms, but the pattern of variation according to environment was similar: mice maintained outdoors harbored greater nematode burdens than Lab mice (S1A Fig; Long-term Wild versus Lab: *z* = 3.367 and *P* = 0.00214; Short-term Wild versus Lab: *z* = 3.032 and *P* = 0.00659; and *P* > 0.9 for comparison of the two wild groups). By 4 weeks p.i., however, there were no significant differences in worm biomass in mice across locations (S1B Fig). Across both time points, all worms collected from infected Lab mice were in the larval stage, whereas 5% (8/160) and 5.9% (16/272) of measured worms from Short-term Wild and Long-term Wild mice, respectively, had matured into adults. No other gastrointestinal worms were found in any of the mice at either end time point.

Taken together, these results indicate that *T*. *muris* hatching, growth, and/or survival, and thus host susceptibility to infection, were enhanced in outdoor environments compared to laboratory conditions. Longer duration of outdoor residence strengthened several of these associations. Environment also partly eroded the susceptibility difference expected for wild-type C57BL/6 versus STAT6-/- genotypes [29,32].

Although moving to the outdoor enclosures induced transient weight loss, the weights of the mice rebounded, such that Long-term Wild mice weighed significantly more than Lab mice at the end of the experiment (|*z*| = 2.91 and *P* = 0.0099) (S2 Fig; S1 Text). Still, differences in nutritional resources between laboratory and outdoor mice could potentially have contributed to the differences in *T*. *muris* susceptibility (S1 Text). We found no significant differences in total protein and leptin levels in blood between infected and uninfected mice across all environments at 3 weeks and 4 weeks p.i. (S3A Fig, S3B Fig, S1 Table). There was, however, a significant location by infection effect on blood albumin at these two time points, with uninfected Lab mice exhibiting the lowest levels of blood albumin (S3A Fig, S3B Fig, S1 Table). Interestingly, this albumin pattern suggests that the wild mice may be at a higher protein nutritional plane than their laboratory counterparts and thus cannot explain the observed differences in *T*. *muris* susceptibility across locations. Instead, we identified two significant correlates of the enhanced nematode susceptibility in outdoor mice: microbial and immunological, as described in the following sections.

### Enriched gut microbiota in mice kept outdoors

**Fig 3:**
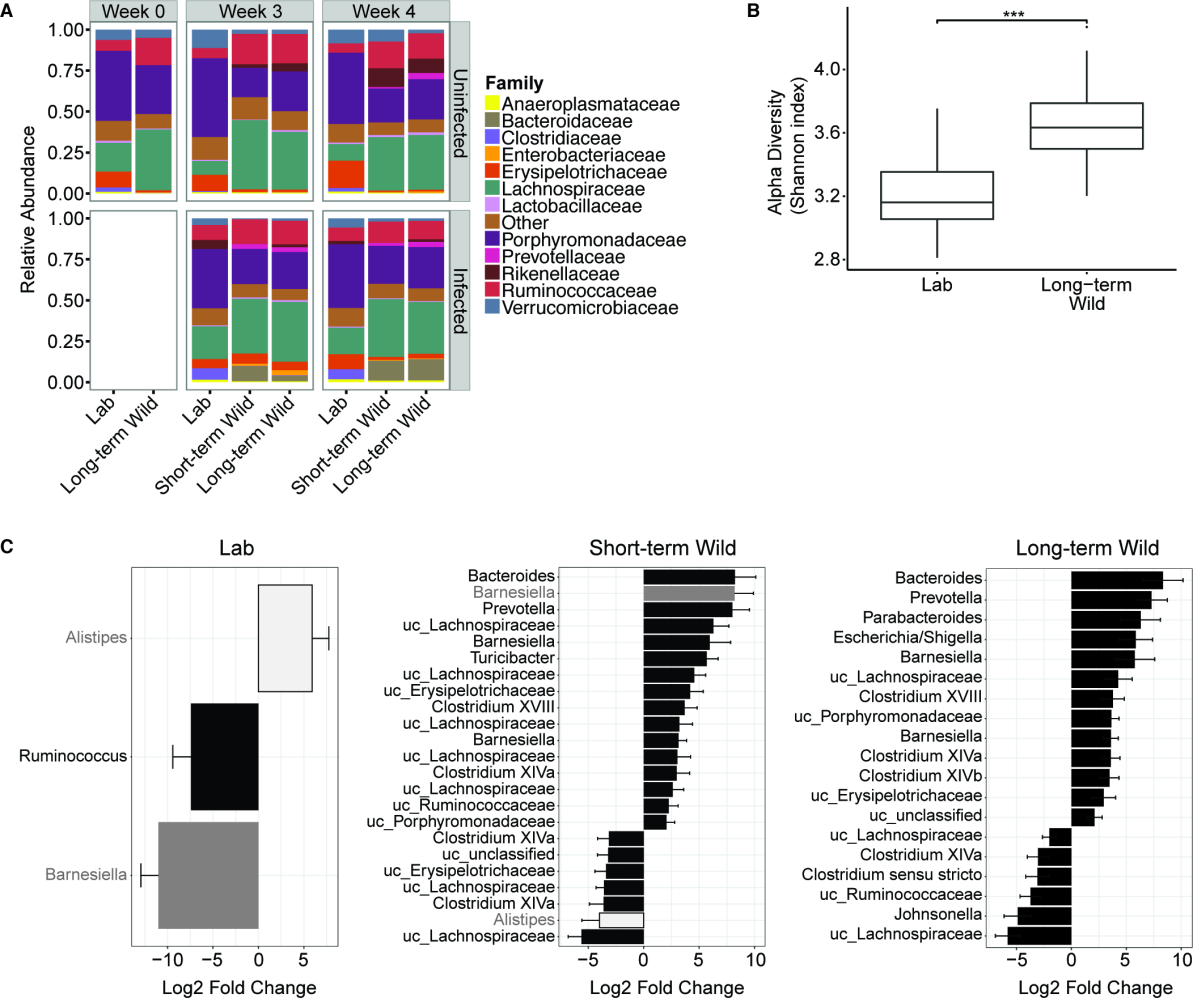
Heterogeneous environments and *Trichuris muris* infection alter the composition of the fecal microbiota. (**A**) Taxa summary plots at the family level showing the microbiota composition of uninfected and infected mice at Week 0 and at Week 3 and Week 4 p.i. Data represent the mean relative abundance. Blank spaces represent an absence of data for those groups. The Lab group at Week 0 includes both Lab and Short-term Wild mice, as both groups were residing under laboratory conditions at this time. (**B**) Shannon diversity of fecal samples from mice residing in laboratory and outdoor environments before *T*. *muris* infection (Week 0). The Lab group includes both Lab and Short-term Wild mice, as both groups were residing under laboratory conditions at this time. Box centers show the medians, and the upper and lower box edges correspond to the 25th and 75th percentiles, respectively. Whiskers extend 1.5 times the interquartile range. The difference between diversity was determined using an unpaired Student *t* test. Asterisks denote significance as ***P < 0.001. (**C**) Mean log_2_ fold change of OTUs in infected compared to uninfected mice residing in laboratory and outdoor environments at 3 weeks p.i. using DESeq2. Data shown have been filtered to include OTUs that have a *P*-adjusted value <0.05 and a log_2_ fold change >|2| with a baseMean >20 to show the abundant OTUs that are most changed with infection. Bars are colored to direct the attention of readers to two main OTUs, *Barnesiella* and *Alistipes*. Gray bars are used to show how *Barnesiella* is decreased in Lab mice but increased in Short-term Wild mice. White bars are used to show how *Alistipes* is increased in Lab mice but decreased in Short-term Wild mice. Bars show mean + standard error. Mice that had purged all worms (Lab mice: *N* = 2) were excluded from the infected group analyses. 16S rRNA gene sequences are available at NCBI SRA: SRP132155. NCBI, National Center for Biotechnology Information; OTU, operational taxonomic unit; p.i., postinfection; uc, unclassified.

Long-term Wild mice resided outdoors for 2 weeks before *T*. *muris* infection (Fig 1B). This period outdoors altered the composition and diversity of their gut microbiota compared to mice residing in the laboratory (Fig 3A top left panel, 3B). Bacteroidetes and Firmicutes made up the top two phyla of the murine gut in both locations (S4A Fig). However, at lower taxonomic levels, residing outdoors for only 2 weeks induced clear shifts in the composition of the gut microbiota. Outdoor environments led to a significant increase in the abundance of operational taxonomic units (OTUs) belonging to the Enterobacteriaceae, Lachnospiraceae, and Ruminococcaceae families and a reduction in OTUs belonging to the Clostridiaceae and Erysipelotrichaceae families, compared to mice residing under laboratory conditions (Fig 3A top left panel, *P* < 0.05). Relocation of Short-term Wild mice from the laboratory to the outdoors similarly shifted their gut microbial community to more closely resemble that of the Long-term Wild mice after just 10 days outdoors (Fig 3A, top right two panels).

The alpha (within samples) and beta (between samples) diversity of the intestinal microbiota also differed among mice residing outdoors for two weeks compared to mice residing in the laboratory. Alpha diversity (calculated for unfiltered data using the Shannon index) was significantly higher in Long-term Wild mice after 2 weeks of outdoor exposure compared to mice that had been residing under laboratory conditions (Fig 3B; *P* < 0.0001). Based on the Bray-Curtis dissimilarity distances, both time (i.e., Week 0 versus Week 3 and Week 4 p.i.) and the environment (i.e., laboratory versus outdoors) also significantly altered the gut community structure and hence the beta diversity of bacteria in laboratory versus outdoor mice (S4B Fig, PERMANOVA test: *P* < 0.05). However, no significant difference in species richness as measured by Chao1 was observed between outdoor and laboratory mice (S4C Fig, *P* = 0.089). We did, however, find approximately a quarter log reduction in bacterial density, as measured by 16S gene copies/μg of DNA, in the Long-term Wild mice compared to mice in the laboratory (S4D Fig, unpaired *t* test: *P* < 0.05). Thus, mice residing in the outdoor enclosures for just 2 weeks harbored an altered gut microbial composition, diversity, and bacterial density compared to mice in the laboratory.

Mice kept outdoors also acquired different fungal communities in their guts compared to mice residing in the laboratory (S4E Fig, top left panel). This was shown most evidently in a randomized subset of the Short-term Wild mice. At Week 0, when the Short-term Wild mice were still residing under laboratory conditions, only 25.0% of mice (5 out of 20 mice) were positive for fungi. After residing outdoors for at least 10 days, 90.0% of the Short-term Wild mice (18 out of 20 mice) now harbored fungal communities. Metabarcoding of fungal communities revealed that the top three fungal families present in the Short-term Wild mice under laboratory conditions (Week 0) belonged to the families Debaryomycetaceae, Mrakiaceae, and Mucoraceae, whereas movement outdoors led to increased abundances of OTUs belonging to the family Chaetomellaceae and a larger proportion of unclassified fungal families (S4E Fig).

At the study end point, serology testing for 18 common pathogens of mice (see Materials and methods) revealed that the Short-term Wild and Long-term Wild mice had not been detectably exposed to infections aside from the inoculated worms. These changes demonstrate that movement of C57BL/6 mice to outdoor environments rapidly altered the composition of the gut microbial community, and while mice acquired new bacterial and fungal communities in the gut, we detected no exposure to common mouse pathogens.

### Realigned gut microbe–nematode communities in mice kept outdoors

Infection with *T*. *muris* induced distinct changes to the gut microbiota, depending on the host environment (Fig 3C, *P* < 0.05). In Lab mice, at 3 weeks p.i., we identified an increase in an OTU belonging to the genus *Alistipes* and a decrease in OTUs belonging to the *Ruminococcus* and *Barnesiella* genera with infection compared to uninfected Lab mice. Once mice moved to the outdoor enclosures, the acquisition of more diverse microbes (Fig 3B) likely enabled a greater number of OTUs to be differentially regulated upon *T*. *muris* infection, as we observed.

Crucially, a few key OTUs altered by nematode infection in the rewilded groups showed opposite trends compared to mice in the laboratory. For example, at 3 weeks p.i., the same OTU belonging to *Alistipes*, which was enriched with worm infection in Lab mice, was reduced in the Short-term Wild infected mice compared to Short-term Wild uninfected mice. Additionally, the same *Barnesiella* OTU, which showed the largest log-fold decrease in laboratory mice due to worm infection, was now one of the most elevated OTUs found in the Short-term Wild group following infection (Fig 3C). At 4 weeks p.i., the presence of this *Barnesiella* OTU was also increased in the Long-term Wild infected mice compared to Long-term Wild uninfected mice (S5A Fig). These results suggest that interactions between *T*. *muris* and the gut microbiota (the main constituents of the gut community here) are likely to be influenced by the environment in which the host lives, and these effects may oppositely alter specific bacterial taxa, depending on the host environment.

Alpha diversity analysis using the Shannon index revealed a significant effect of infection, location, and the interaction between infection and location at 3 weeks p.i. (S5B Fig; S2 Table). Short-term Wild and Long-term Wild uninfected mice had increased alpha diversity compared to uninfected and infected Lab mice. There was also a decrease in Shannon diversity due to infection in the Short-term Wild mice. Additionally, there was a significant main effect of location on bacterial density (*P* = 0.0001), with up to half a log reduction in bacterial density in uninfected Long-term Wild mice compared to Lab mice (S5C Fig), which was a similar trend to what was observed at Week 0 (S4D Fig). At 4 weeks p.i., there was a significant effect of location on Shannon diversity (S2 Table). Beta diversity using Bray-Curtis dissimilarities revealed a greater dissimilarity among samples from different locations and due to time than with nematode infection (S4B Fig; PERMANOVA test: P<0.05). These indices demonstrate that although nematode infection impacts gut microbial diversity, the impacts of environment and time, especially outdoors, are considerably stronger.

### Decreased Th2 and increased Th1 bias in *T*. *muris* infected mice kept outdoors

Lamina propria mononuclear cells (LPMCs) from the colon were isolated from a randomized subset of infected and uninfected mice to evaluate gut mucosal responses to *T*. *muris* infection under varying environments. Among these, we focused primarily upon phenotyping the CD4+ T (i.e., “T-helper”) cells due to their established role in determining susceptibility to gastrointestinal nematodes [41], but we also collected data on phenotypes of the CD8+ T (i.e., “killer T”) cells (S6A Fig, see below). To characterize wider, intestinal immune phenotypes of the mice, we also cultured mesenteric lymph node (MLN) cells in vitro and measured production of cytokines.

**Fig 4:**
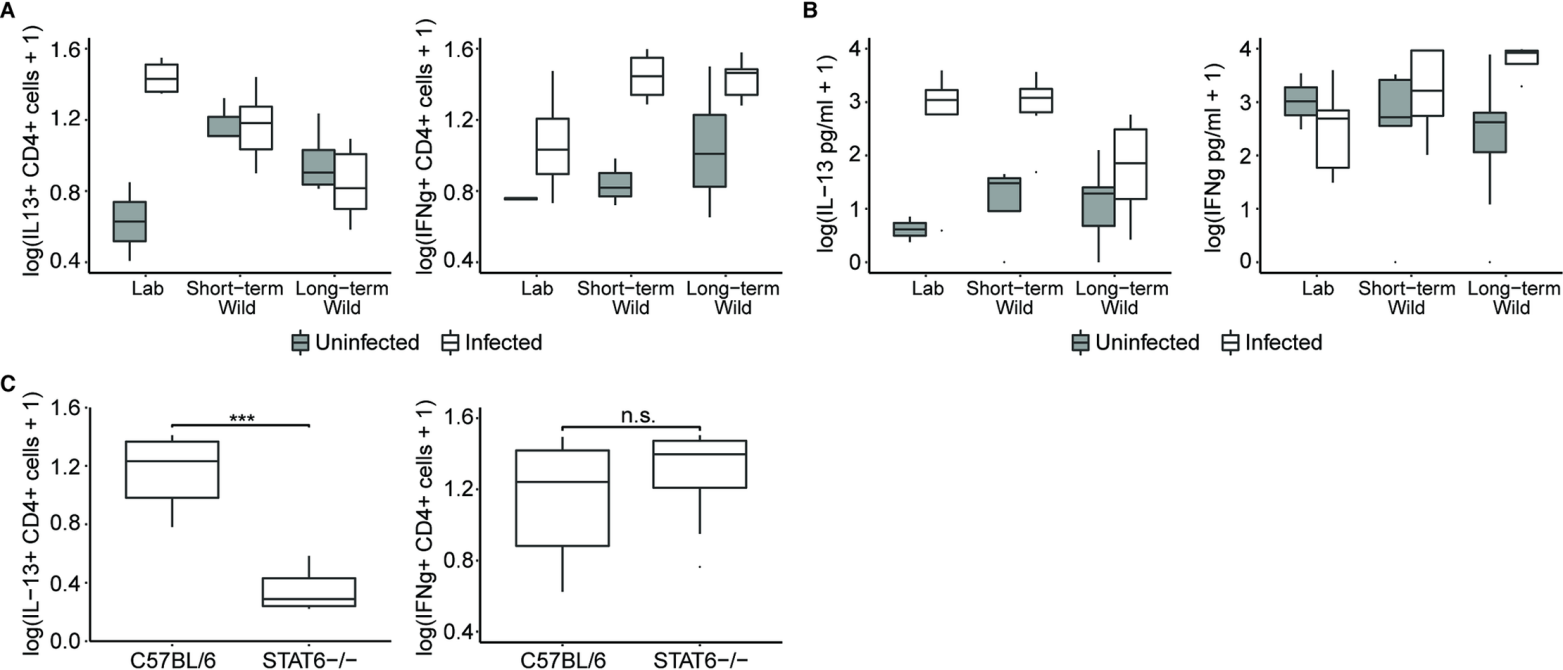
Decreased Th2 and increased Th1 cytokines in LPMCs and MLNs from *Trichuris muris–*infected mice residing outdoors compared to laboratory conditions at 3 weeks p.i. (**A**) Proportion of CD4+ cells that are producing IL-13 and IFNγ from LPMCs of mice residing in laboratory and outdoor environments at 3 weeks p.i. Sample sizes for that time point were chosen following power calculations using pilot data: Uninfected Lab mice: *N* = 2; Infected Lab mice: *N* = 4; Uninfected Short-term Wild mice: *N* = 3; Infected Short-term Wild mice: *N* = 5; Uninfected Long-term Wild mice: *N* = 4; Infected Long-term Wild mice: *N* = 6. (**B**) Concentrations of IL-13 and IFNγ produced from MLNs of mice residing in laboratory and outdoor environments 3 weeks p.i. Sample sizes: Uninfected Lab mice: *N* = 2; Infected Lab mice: *N* = 8; Uninfected Short-term Wild mice: *N* = 5; Infected Short-term Wild mice: *N* = 10; Uninfected Long-term Wild mice: *N* = 9; Infected Long-term Wild mice: *N* = 10. (**C**) Proportion of CD4+ cells that are producing IL-13 and IFNγ from LPMCs of infected C57BL/6 (*N* = 4) and STAT6-/- (*N* = 5) mice residing outdoors for the short term at 3 weeks p.i. Data on cytokine-positive lamina propria cells and in vitro cytokine secretion were log(x+1) transformed to meet assumptions of analysis. Box centers show the medians, and the upper and lower box edges correspond to the 25th and 75th percentiles. Whiskers extend 1.5 times the interquartile range. https://doi.org/10.5061/dryad.h9g697r. IFNγ, interferon-gamma; IL, interleukin; LPMC, lamina propria mononuclear cell; MLN, mesenteric lymph node; p.i., postinfection; STAT6-/-, mouse deficient in STAT6; Th1, type 1; Th2, type 2.

For many of these immunological readouts, we found significant interactions between mouse location and infection (S3 Table: At 3 weeks p.i., significant interaction terms for cytokines in LPMCs include IL-13, IL-4, and IL-17, and for MLN cells, IL-13, IFNγ, and IL-10), suggesting that the mice responded differently to nematode infection in the laboratory versus field. Changes to the Th2 and Th1 balance of the immune response when outdoors were of particular interest, as follows. At 3 weeks p.i., infected Lab mice exhibited the expected increase in the proportion of T-helper cells producing IL-13 compared to uninfected Lab mice (Fig 4A, S3 Table). However, mice kept outdoors had higher baseline proportions of IL-13–producing cells (in uninfected mice) and lower induction of IL-13 (in infected mice) than their laboratory-kept counterparts. For example, infected Long-term Wild mice residing outdoors had significantly decreased proportions of cells producing IL-13 in CD4+ LPMCs compared to infected Lab mice (Fig 4A; S3 Table; two-way ANOVA interaction effect: F(2,18) = 11.20; *P* = 0.0007). These IL-13 differences were also mirrored in MLNs stimulated against *T*. *muris* antigens at 3 weeks p.i. (Fig 4B; S3 Table; two-way ANOVA interaction effect: F(2,38) = 3.65; *P* = 0.035). Conversely, there was a trend towards an increased proportion of IFNγ-producing CD4+ LPMCs in the two infected wild groups compared to Lab mice (Fig 4A; S3 Table; baseline levels did not differ significantly). This difference was also reflected in MLNs, with infected Long-term Wild mice producing significantly more IFNγ than infected Lab mice (Fig 4B; S3 Table; two-way ANOVA interaction effect: F(2,38) = 3.68; *P* = 0.035). Interestingly, while infected STAT6-/- were indeed deficient in the proportion of CD4+ cells producing IL-13 in LPMCs (Fig 4C), the proportions of CD4+ IFNγ+ cells were indistinguishable between infected C57BL/6 and STAT6-/- mice after just 10 days outdoors (Fig 4C; *P* > 0.05). Aside from CD4+ IL-4+ production in LPMCs, which followed trends similar to that of IL-13, production of other CD4+ cytokines in LPMCs and MLNs did not show such clear patterns in relation to location or infection status (S6B Fig, S6C Fig, S1 Text). Furthermore, at 3 weeks p.i., only IFNγ and tumor necrosis factor-alpha (TNFα) were produced by CD8+ T cells in the lamina propria, with a highly significant increase in the proportion of CD8+ cells producing IFNγ associated with nematode infection (i.e., the mice dosed with nematode eggs) across host environments (S6D Fig; S3 Table; *P* < 0.0001).

At 4 weeks p.i., the differences in the proportion of CD4+ cells producing IL-13 in LPMCs due to a main effect of location disappeared, but there was still a significant effect of infection and an interaction between location and infection (S7A Fig, S3 Table: At 4 weeks p.i., significant interaction effects for LPMC cytokines include IL-13, IL-4, IL-17, and TNFα). There were also significant main effects of location and infection upon IL-13 production in MLNs (S7B Fig, S3 Table). Long-term Wild mice also had significantly increased proportions of CD4+ cells producing IFNγ in LPMCs compared to Lab mice (S7A Fig, S3 Table). IFNγ and TNFα were again the only cytokines produced by a significant proportion of CD8+ T cells in the lamina propria at the Week 4 time point, with an increase in the proportion of CD8+ cells producing IFNγ among mice that had resided outdoors longest (S7C Fig; S3 Table; *P* = 0.018).

**Fig 5:**
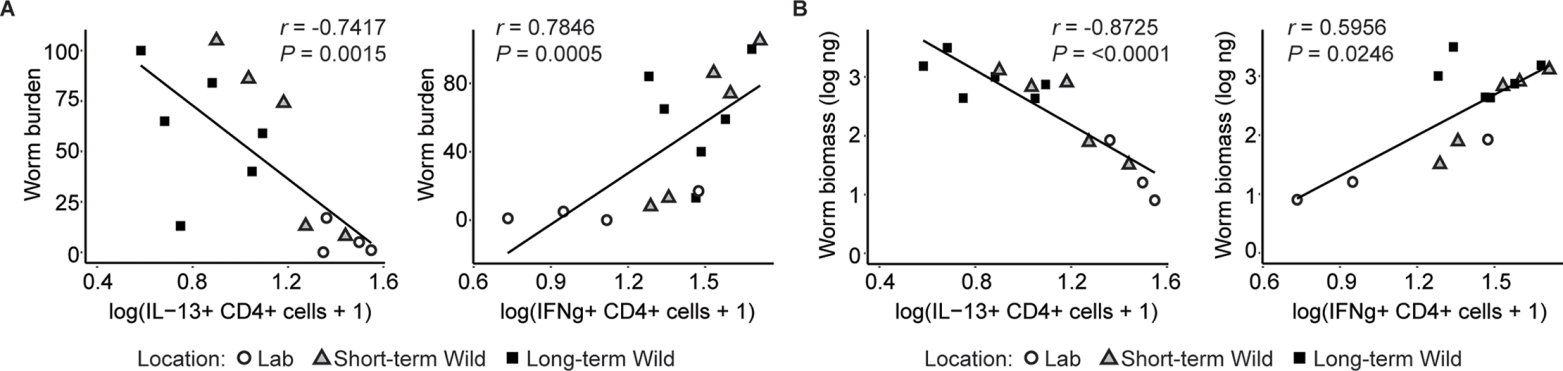
High worm burdens and worm biomass are associated with decreased proportions of Th2 and increased proportions of Th1 cells in LPMCs of *Trichuris muris–*infected mice. (**A**) A Spearman’s correlation was performed between worm burden and the proportion of CD4+ cells that are producing IL-13 and IFNγ from LPMCs of infected mice in the laboratory and outdoor enclosure at 3 weeks p.i. (**B**) A Spearman’s correlation was performed between worm biomass and the proportion of CD4+ cells that are producing IL-13 and IFNγ from LPMCs of infected mice in the laboratory and outdoor enclosure at 3 weeks p.i. Data on cytokine-positive lamina propria cells were log(x+1) transformed to meet assumptions of analysis. These correlations were all significant (*P* < 0.05) with *P* values and *r* values shown on graphs. White circles = Lab; gray triangles = Short-term Wild; black squares = Long-term Wild. https://doi.org/10.5061/dryad.h9g697r. IFNγ, interferon-gamma; IL, interleukin; LPMC, lamina propria mononuclear cell; p.i., postinfection; Th1, type 1; Th2, type 2.

These results indicate that the Th1 and Th2 immune responses of CD4+ LPMCs and of MLN cells responding to *T*. *muris* antigen at 3 weeks and 4 weeks p.i. are significantly altered by the environment of the host. Unsurprisingly, the dampened Th2 and elevated Th1 response was associated with increased mean susceptibility in the mice kept outdoors. Indeed, among infected mice in the laboratory and outdoors, correlational analyses between worm burdens and the CD4+ LPMC cytokine data at 3 weeks p.i. revealed that higher worm burdens were significantly associated with decreased levels of IL-13–producing CD4+ cells and increased levels of IFNγ-producing CD4+ cells (Fig 5A; Spearman's rank correlation coefficient, all *P* < 0.05) across treatment groups. Similarly, worm biomasses at 3 weeks p.i. were associated with decreased proportions of CD4+ cells producing IL-13 cells and increased proportions of CD4+ cells producing IFNγ (Fig 5B; Spearman's rank correlation coefficient, all *P* < 0.05). LPMC analysis also confirmed that the Th2 deficiency of STAT6-/- mice was maintained outdoors (Fig 4C, S8 Fig), suggesting that the IFNγ bias of all outdoor mice may be the most important immunological driver of their nematode susceptibility. Regression analysis using a random forest algorithm showed no significant association between the variation in the proportion of CD4+ cells producing IL-13 or IFNγ and the presence of microbial or fungal families. Taken together, these results demonstrate that residing outdoors skews the type of immune response elicited against *T*. *muris* infection from a Th2-dominated response observed in the laboratory to a Th1 response, which likely impacts worm burden and biomass.

## Discussion

We investigated how changes to the external environment can lead to phenotypic variation in the susceptibility of a given host genotype to the gastrointestinal helminth, *T*. *muris*. We found that the C57BL/6 mouse strain, which is resistant to *T*. *muris* infection in the laboratory [42], interacts differently with nematodes when exposed to an outdoor environment. Outdoor mice experienced increased susceptibility to *T*. *muris* infection, exhibited by increased worm burdens and worm biomass, compared to mice residing under clean laboratory conditions. These changes in susceptibility were observed after just 10 days outdoors, as demonstrated in the Short-term Wild group, revealing how rapidly nematode susceptibility can change with environment. The environment-induced changes in worm burden and worm biomass in C57BL/6 hosts persisted to 4 weeks p.i., although how long the effects would have lasted thereafter is unknown. At a minimum, the new environment prolonged the persistence of large worms for several weeks in this otherwise resistant host genotype. We note that our maintenance of Lab mice at field-mimicking temperature and humidity conditions (which arguably brings mice closer to their thermal and thus physiological optima [25]) did not render them susceptible to *T*. *muris*.

We primarily investigated two potential ecological mechanisms that could be capable of explaining these alterations in susceptibility: environmental alterations to the gut microbiota and the host immune response, which may interact with one another to impact different aspects of worm life history and produce the observed effects. We expected these changes to alter nematode susceptibility via positive or negative effects on worm hatching, growth, and/or survival. We expected worm hatching would be determined by microbial diversity and composition, whereas worm growth and survival would depend on both microbial and immune factors. Indeed, by manipulating the time point at which worms interacted with the gut microbiota, we showed that environmental impacts on multiple worm stages combined to enhance the nematode susceptibility profiles of rewilded mice.

First, the increased diversity and novel composition of microbial communities resulting from residence outdoors could alter *T*. *muris* hatching, colonization, and growth, thus contributing to the differences observed in worm burdens between groups. Indeed, the gut microbial community is known to play an essential role in host colonization by *T*. *muris* [26,27]. Eggs of the nematode can only hatch when gut microbes directly attach to the polar egg caps, and depleting gut bacteria with antibiotics significantly reduces *T*. *muris* hatching rates in mice [26]. This requirement for the gut microbiota appears to be shared among helminth species, as germ-free (microbiota-lacking) and gnotobiotic hosts exhibit increased resistance to a variety of helminths [43–47]. In our study, two weeks of outdoor exposure prior to *T*. *muris* infection increased gut microbial diversity and altered the composition of the gut microbiota in Long-term Wild mice, compared to mice residing under laboratory conditions. The rapidity of microbial changes is not unexpected, given (for example) how quickly diet changes can alter the human microbiota [48]. The enhanced microbial diversity in the Long-term Wild mice prior to *T*. *muris* infection may have contributed to higher hatching rates of nematodes and, hence, higher worm burdens compared to mice in the laboratory. We also observed a modest, yet significant, quarter log reduction in bacterial density in outdoor mice compared with laboratory mice. This difference could be attributable to microbe turnover in a new environment, such that certain preexisting bacteria may be rapidly lost in the outdoor environment and only slowly replaced by new taxa. We note that our observed reduction in bacterial density is certainly less than that observed with antibiotic treatment, which showed significant effects on *T*. *muris* worm burdens [26]. Future studies with more frequent sampling time points could help elucidate the successional dynamics of microbes, in terms of both density and diversity, in the rewilded mice.

The presence of particular gut microbes can also facilitate the persistence of helminth infections, whether directly, such as nematodes grazing on gut flora, or indirectly, via immunomodulation. For instance, certain species of *Lactobacillus* have been shown to promote the persistence of *T*. *muris* [49] and *H*. *polygyrus* [50] worm burdens in specific mouse strains. Previous studies investigating alterations in the gut microbiota during *T*. *muris* infection [51,52] found similar gut microbial changes as seen in our Lab mice. For instance, Holm et al. (2015) also observed increased abundances of *Barnesiella* and decreased abundances of *Alistipes* with low-dose *T*. *muris* infection in C57BL/6 laboratory mice [51]. Our results indicate that OTUs in these two taxa were oppositely abundant in mice residing outdoors, although the changes were not entirely consistent across all groups and time points. However, the reversal of the abundances of *Barnesiella* and *Alistipes* and the additional correlation with *Ruminococcus*, all of which we observed during nematode infection in outdoor environments, could be critical factors impacting the survival of *T*. *muris* in the murine gut and warrant further testing.

The second major mechanism that we investigated as potentially driving the differences in *T*. *muris* susceptibility was the host immune response following changes to the environment. For *T*. *muris*, it is well established that resistance and susceptibility are tightly associated with the generation of a Th2 or Th1 immune response, respectively [53]. Our results indicate that residing outdoors skews the host immune response towards a Th2 response at baseline, but then towards an induced Th1 response during *T*. *muris* infection. Given that outdoor mice harbored increased fungal communities and that most fungi induce a Th2 response [54], we tested whether the increase in Th2 responses at baseline were due to fungi, but no association between the strength of the Th2 response and fungal abundance was found. As for the induction of IFNγ during nematode infection, this could result from the introduction of new microbes into the gastrointestinal tract of mice (as described above) and/or be due to higher overall immune activation rates (both Th1 and Th2) outdoors [13]. In *T*. *muris–*susceptible animals, IFNγ has been shown to play a critical role in regulating the processes underlying epithelial cell turnover [55], which is an efficient and effective mechanism of *T*. *muris* expulsion from the gut [28]. Production of IFNγ induces the chemokine C-X-C motif chemokine ligand10 (CXCL10), which slows down the rate of epithelial cell turnover required to expel *T*. *muris*, and expulsion is especially likely to fail once the nematodes achieve a threshold size [28]. Microbe-facilitated growth of the nematodes and Th1-skewed immunity might therefore synergize to enhance host susceptibility. In fact, IFNγ production and *T*. *muris* worm burdens in wild-type mice kept outdoors for the short term were nearly as high as those of STAT6-/- mice that are genetically susceptible to nematode infection. Despite similar worm burdens between STAT6-/- and wild-type mice, the significantly higher worm biomass in STAT6-/- mice supports the important role of host immunity in worm growth. While previous studies found an increase in highly differentiated CD8+ T cells in blood samples from wild compared to laboratory mice [17], we observed no difference in the proportion of CD8+ T cells in LPMCs of our laboratory versus outdoor mice, although there was an increase in IFNγ positivity in CD8+ cells among mice that had resided longest outdoors at 4 weeks p.i. Thus, the new environment rapidly altered host responses to parasitism through alterations to both the gut microbiota and immune response.

Interestingly, Rosshart et al. (2017) recently demonstrated increased resistance to viral influenza infection in laboratory mice reconstituted with the natural microbiota from wild mice [15]. In their study, the wild mouse gut microbiota abrogated excessive inflammation and inflammatory cell infiltration, which was beneficial in promoting host fitness in the context of a lethal influenza infection. Increased resistance to infection is what may be expected for mice made more immunologically competent following natural microbial exposure (e.g., [17]). However, our study demonstrates that helminth-infected mice exposed to a more natural microbial environment actually experienced increased susceptibility to nematode infection, due in part to their induction of Th1 responses with infection. This result would be expected given the Th2 dependence of worm resistance. Future studies investigating how exposure to a more natural microbial environment alters susceptibility to microparasites compared to macroparasites are thus warranted.

The intestinal environment that the worms experienced in the Lab, Short-term Wild, and Long-term Wild mice were ultimately quite different due to the relative timing of nematode inoculation and the movement outdoors. This allowed us to assess the impacts of microbial and immune factors on different life stages (e.g., hatching, growth, and survival) of *T*. *muris*. The Lab mice had low gut microbial diversity at worm hatching and were able to mount an effective Th2 response following infection. The Short-term Wild mice had low gut microbial diversity at worm hatching and potentially impaired immunocompetence while responding to the growing worms. The Long-term Wild mice, by contrast, harbored diverse gut microbes at worm hatching (a potential driver of high nematode colonization) but also high baseline Th2 immunity (a potential driver of low nematode colonization). At 3 weeks p.i., the average worm burdens in the Long-term Wild mice were over 20 times those of the Lab mice. The Short-term Wild mice also harbored over 13 times as many worms as the Lab mice on average. Our study design cannot conclusively distinguish between increased helminth hatching or reduced expulsion rates in generating the observed worm burden differences. We can nonetheless infer the following. Given the lack of difference in worm burdens between the Short-term Wild mice, who were initially infected in the laboratory, and the Long-term Wild mice, we can deduce that an increased hatching rate is not the only explanation for the higher worm burden observed outdoors. Moreover, because the Short-term Wild mice and Lab mice were infected under the same laboratory conditions and hence should have comparable rates of egg hatching, we can deduce that Lab mice effectively expelled their worms by 3 weeks p.i., whereas the worms in the Short-term mice survived longer following their host’s movement to an outdoor environment.

Furthermore, the total worm biomass significantly differed between the Lab, Short-term Wild, and Long-term Wild mice. The average log biomass per worm in the Long-term Wild mice was over three times greater than that of the Short-term Wild mice and over seven times greater than that of the Lab mice. The worms in the Short-term Wild mice also had over twice the average log biomass of worms in the Lab mice. The larger per-worm biomass of worms in mice residing longest outdoors suggests that the environment accelerated growth and generated more robust worms. Together, our results show that enhanced worm growth or survival, and not just enhanced hatching, must be critical factors in determining *T*. *muris* susceptibility of rewilded mice.

Host environment during nematode inoculation could also potentially affect nematode colonization via host energetic or other stressors, which are potentially immunosuppressive [56]. However, our results suggest that such factors cannot fully explain the observed differences in nematode susceptibility across groups. The relocation outdoors coincided with a transient period of weight loss, potentially due to increased energetic demands (e.g., exercise) or stress associated with the move. The Short-term Wild mice moved outdoors and experienced a temporary weight loss period while they were already infected with nematodes. The Long-term Wild mice, on the other hand, had recovered their body weight and had at least acclimated to some of the stresses of the enclosure by the time of nematode inoculation. Thus, the Long-term Wild mice may have been nutritionally and otherwise better able to mount an immune response to worms, as demonstrated by their increased concentrations of albumin compared to uninfected Lab mice. However, we observed that the Long-term Wild mice were in fact more susceptible to worm infection, as the worms they harbored were significantly larger in size compared to the Short-term Wild mice. Less hatching, but also less immune pressure, could have led to the intermediate worm burdens and worm biomass observed in Short-term Wild mice. It is important to note that while the rewilded mice were able to access food sources found within the enclosures, including berries, seeds, and insects, they were also provided ad libitum with the same mouse chow used in the laboratory, and thus their nutritional state may differ from that of truly wild mice.

Others have shown that host susceptibility to helminth infection is altered in more natural arenas [21,22]. For example, strains of mice defined in the laboratory as differentially susceptible to *H*. *polygyrus* infection become indistinguishable under low transmission rates [21,22]. Similarly, different infective doses of *H*. *polygyrus* alter the fitness cost paid by hosts, with resistant mouse strains paying the highest costs with increasing parasite exposure [23]. However, these helminth studies were all conducted in indoor cages or arenas with mice maintained on controlled substrates that were inoculated with uncontrolled doses of nematode larvae. Our study is the first to show altered susceptibility when nematode exposure is controlled while other aspects of the environment are altered. We provided a more realistic environment outdoors to *M*. *musculus*, with natural vegetation, weather, and microbial exposure as experienced in the wild. Furthermore, in these previous helminth studies, the within-host mechanisms underlying these changes in susceptibility were not examined. We sought to uncover such mechanisms by examining associations of the gut microbial community and host immune response with susceptibility differences to equivalent *T*. *muris* exposures in laboratory and outdoor mice. In order to prevent transmission cycles and compare with the worm expulsion dynamics of C57BL/6 mice in the laboratory, we ended our experiment before *T*. *muris* fully developed into adults (expected 32 days p.i. [57]). Natural transmission of *T*. *muris* would have also required approximately 2 months for released eggs to embryonate and become infective [58]. In future, longer-term studies, it would be interesting to investigate whether the entire life cycle of *T*. *muris* progresses faster and with greater fecundity in outdoor versus laboratory conditions and to assess host susceptibility under natural transmission at uncontrolled doses.

Previous studies in other host taxa have shown that small yet realistic changes in the environment can significantly alter host responses to parasitism. For instance, slight increases in temperature can cause genotypes of the freshwater crustacean, *Daphnia magna*, to reverse their susceptibility profiles against bacterial pathogens [8]. Similar genotype-by-temperature interactions (e.g., [59–61]) as well as genotype interactions with biotic factors such as population density, food sources, predation, and competition (e.g., [62–64]) alter host susceptibility to infection in a range of other invertebrates. These studies demonstrate that the environment can maintain variation in immune defense by favoring certain host genotypes over others, depending on the environment in which the host lives. Our study, although not a factorial genotype by environment (GxE) design, shows the profound impact of environment on the rates at which nematodes are established or cleared from mammalian hosts of a controlled genotype. Heterogeneous environments can thus shape the variation observed in mammalian susceptibility to infection and potentially the evolutionary trajectories of host and parasite alike.

In summary, our data demonstrate that environment has a clear and rapid effect on host susceptibility to nematode infection, enhancing susceptibility of C57BL/6 mice that are highly resistant under laboratory conditions. We observed multiple changes in the gut microbial community and intestinal immunity as a result of the movement to outdoor enclosures. Our experimental manipulation of the time point at which the worms and microbes began to interact suggests that impacts on multiple worm life stages contribute to the differences observed in *T*. *muris* susceptibility in the rewilded mice. Furthermore, natural environments, such as our outdoor enclosures, provide the opportunity for mice to compensate energetically for infection and induced defense with additional or selective feeding [24]. Thus, laboratory studies conducted under a single environment, while important for uncovering detailed, molecular mechanisms of defense, are likely to illuminate only a small fraction of the causes of heterogeneity in nematode burden, especially given the immense differences in immune phenotypes of laboratory and wild mice [17] and the substantial immune divergence between *M*. *musculus* strains in the laboratory versus the field [13]. Just as for genetic variation [65], we suggest that future empirical studies therefore need to incorporate diverse environments to better reflect the context-dependency of host susceptibility and resistance in the wild [66]. Such studies will elucidate how the external environment impacts the ecology of symbioses and defenses within.

## Materials and methods

### Ethics statement

All experimental procedures were approved by the Princeton University Institutional Animal Care and Use Committee (Protocol #1982–14).

### Animals, outdoor enclosures, nematode exposures, and sampling

Specific pathogen-free female C57BL/6 mice (6–8 weeks of age) were obtained from Jackson Laboratories. Upon arrival, animals were housed in cages in groups of 5 after insertion of ear tags and radio-frequency identification (RFID) transponders to identify individuals. Mice were gradually acclimated to and then kept for 2 weeks at 26°C ± 1°C with a 15-hour light/9-hour dark cycle (to approximate local summer solstice daylight patterns during the May–July experimental time frame). After 1 week, cage bedding was swapped with a bedding mixture from all of the cages to homogenize the gut microbiota. Cages were then randomly assigned to one of three environment groups: Lab (*N* = 20), Short-term Wild (*N* = 30), and Long-term Wild (*N* = 40). Mice within each environment were then randomly assigned to infection groups. Lab mice, housed in cages in groups of 5, remained in the laboratory with the above temperature and light conditions for the duration of the study. Short-term Wild mice remained in the above laboratory conditions initially and then moved to the outdoor enclosures 10 days after infection with *T*. *muris*. Long-term Wild mice resided in outdoor enclosures for the duration of the study, receiving the nematode inoculum after 2 weeks outdoors (Fig 1B).

The enclosures consist of replicate outdoor wedge-shaped pens arranged in a circle, each measuring about 180 m^2^ and fenced by a 1.5-m high, zinced iron wall that is buried >80 cm deep and topped with electrical fencing to keep out terrestrial predators. Aluminum pie plates were strung up to deter aerial predators. A small (180 × 140 × 70 cm) straw-filled shed was present in each enclosure, along with two watering stations and a feeding station, so that the same mouse chow used in the laboratory (PicoLab Rodent Diet 20) was provided ad libitum to all mice. Mice outdoors, however, also had access to food sources found within the enclosures, including berries, seeds, and insects. Each enclosure housed 11–12 mice. Short-term Wild and Long-term Wild mice were released into the same enclosures but separated by infection status (i.e., 4 enclosures for infected mice and 2 enclosures for uninfected mice); cages were otherwise randomly assigned to enclosures. Longworth traps baited with chow were used to catch mice weekly; approximately two baited traps were set per mouse per enclosure in the early evening, and all traps were checked within 12 hours. For subsequent microbiome assessment, a fresh stool sample was collected directly from the caught mice, flash frozen on dry ice, and stored at −80°C until further analysis. Mice were also weighed with a spring balance.

Mice assigned to receive nematodes were infected by oral gavage with *T*. *muris* strain E with a high-dose infection using 200 embryonated eggs (as previously described by [57]; see sample sizes in Fig 1B and 1C). Three weeks (days 20–22) p.i. and 4 weeks (days 26–29) p.i., mice were trapped and sampled, as described above. Outdoor mice were then moved to the laboratory overnight and then humanely killed by CO_2_ inhalation followed by cervical dislocation. The cecum and MLNs were removed, and a randomized subset of mice in each treatment group had their colons removed for analysis of LPMCs by flow cytometry.

A second experiment was conducted to compare nematode susceptibility in C57BL/6 mice residing outdoors for the short term with STAT6-/- mice that are highly susceptible to helminth infection. STAT6-/- mice on a C57BL/6 background were originally obtained from Jackson Laboratories and bred at the New York University (NYU) mouse facility. For this experiment, C57BL/6 mice bred at NYU were used for comparison against the NYU-bred STAT6-/- mice. Ten female STAT6-/- mice and 10 female NYU-bred C57BL/6 mice (all 6–8 weeks of age) were acclimated to field conditions in the animal facility at Princeton University. Upon arrival, mice followed the same time line as the Short-term Wild group (Fig 1C), as described above, except that all mice in this experiment were trapped and sampled at 3 weeks p.i. for the end time point. All infected (*N* = 5 of each genotype) and uninfected (*N* = 5 of each genotype) STAT6-/- and C57BL/6 mice were moved to the outdoor enclosures 10 days p.i. STAT6-/- mice were released into separate enclosures based on infection status, and the C57BL/6 mice moved into enclosures with the previous Jackson mice, based upon infection status.

### Worm burden and biomass assessment

The cecum was removed from all infected and uninfected mice at necropsy and frozen at −20°C until assessment of worm burdens. Enumeration of *T*. *muris* worms in the cecum was carried out as previously described [57] (S1 Text). The life stage of each extracted worm was noted based on morphology. Worms were then placed in 100% ethanol for calculation of worm biomass.

To estimate biomass, images of each collected worm were taken under a dissecting microscope using a Canon EOS Rebel T3i and the Adobe Lightroom 5 program. Up to 25 images of different intact helminths from each mouse were randomly selected for measurement of worm length and width using the program, Fiji [67], to calculate the cylindrical volume of each helminth. Given that most of the worms extracted were larvae, a single cylinder appropriately characterizes their volume. For adult worms, the posterior and anterior ends of the worms were measured separately and the sum of the two cylindrical volumes was then calculated for each end. The volume was then multiplied by the assumed wet mass density of 1.1 g/mL for parasites [68] to calculate worm biomass. This assumed density has been found to work well for worms ranging from trematodes to cestodes [69]. To estimate the total helminth biomass per mouse, the mean helminth biomass across the measured worms was then multiplied by the total helminth burden in that mouse. Mice that had purged all worms were excluded from biomass analyses.

### Nutritional measurements

Plasma albumin concentrations were measured colorimetrically using the QuantiChrom BCG albumin assay kits (BioAssays) according to the manufacturer’s instructions. Samples were diluted 1:2 with ultrapure water and run in duplicate. Concentrations were determined in comparison to a standard curve run in duplicate (R^2^ > 0.96). Plasma total protein concentrations were analyzed using the Pierce Coomassie Plus assay kit according to the manufacturer’s instructions. Concentrations were determined in comparison to a standard curve run in duplicate (R^2^ > 0.98). Plasma leptin concentrations were analyzed using a RayBio Mouse Leptin ELISA kit according to the manufacturer’s instructions (RayBiotech). Samples were diluted 1:10, and concentrations were determined in comparison to a standard curve run in duplicate (R^2^ > 0.99).

### 16S rRNA sequencing and analyses

DNA from frozen fecal samples collected at Week 0 (right before *T*. *muris* infection) and at the end of the experiment (either Week 3 or Week 4 p.i.) was extracted using the NucleoSpin Soil Kit (Macherey-Nagel) according to the manufacturer’s instructions. A single fecal pellet per mouse per time point was used for extraction. All DNA samples were shipped to the Research Technology Support Facility at Michigan State University for 16S rRNA sequencing. The V4 region of the 16S rRNA gene was amplified with the universal primers 515F and 806R and sequenced on an Illumina MiSeq sequencer as previously described [70] to generate 2x250 bp paired-end reads. Generated sequences were analyzed using the mothur pipeline [71] (S1 Text), and subsequent analyses were performed in R and R-Studio using the PhyloSeq [72], DESeq2 [73], vegan [74], and GGplot2 [75] packages. DESeq2 was used to detect OTUs that were differentially abundant between compared groups, as previously recommended [73,76]. To identify significantly different OTUs, data were subsetted by sampling time, and a Wald test approach was used with models incorporating infection status and location as predictors. OTUs with adjusted *P* values <0.05, an estimated fold change >2 or <2, and an estimated base mean >20 were considered significantly differentially abundant between the examined groups. To determine if the microbial communities in outdoor mice contributed to the variance observed in Th1 and Th2 immune responses, the random forest algorithm using the R package “randomForestSRC” [77] was applied to the family relative abundance table, using the log(x+1) cytokine responses as the response variable.

Alpha diversity analysis was performed on unfiltered data using the Shannon index. At Week 0, an unpaired Student *t* test was used to detect any significant differences in alpha diversity (as measured by the Shannon diversity index) and species richness (as measured by Chao1) between groups. At 3 weeks and 4 weeks p.i., predictor variables of infection, location, and their interactions were used in models of alpha diversity using a two-way ANOVA, followed by the Tukey post hoc test for multiple comparisons. Beta diversity was analyzed using the Bray-Curtis dissimilarity distance. Due to the highly skewed distribution of bacterial species, a permutation-based multivariate ANOVA (PERMANOVA) [78] using the function “adonis” with 1,000 permutations in the vegan R package [74] was used to analyze beta diversity differences based on time, location, and infection.

### Determination of bacterial density in stool

DNA extracted from fecal samples collected at Week 0 and Week 3 was used for qPCR assessment of bacterial density using the 16S rRNA gene primers for Eubacteria, UniF340 (5-ACTCCTACGGGAGGCAGCAGT-3), and UniR514 (5-ATTACCGCGGCTGCTGGC-3) [79] (S1 Text). To create a standard curve, the 16S gene, obtained from Clostridia strains isolated from stool, was cloned into a pcr2.1-TOPO plasmid, and the DNA was extracted. The amount of extracted DNA and the length of the plasmid were used to calculate the number of 16S copies/μl. Fivefold dilutions of the plasmid standard were then used for quantification of 16S gene copies in the tested samples. 16S gene copies were normalized against the amount (micrograms) of fecal DNA extracted in order to account for the amount of initial stool input. qPCR reactions were performed using the PowerUp SYBR Green Master Mix in a total reaction volume of 20 μl (S1 Text).

### Metabarcoding of fungal communities

DNA from frozen fecal samples was extracted using the NucleoSpin Soil Kit (Macherey-Nagel) according to the manufacturer’s instructions. The internal transcribed spacer (ITS) 1 rDNA region was PCR amplified with the primers ITS5 [80] and ITS5.8 [81] to determine the presence of fungi in the extracted DNA samples. Samples with a positive ITS band were sequenced on a MiSeq and analyzed using OBITools [82] to determine the composition of fungal communities in the fecal samples. To determine if the fungal communities in outdoor mice contributed to the variance observed in Th1 and Th2 immune responses, the random forest algorithm using the R package “randomForestSRC” [77] was applied to the family relative abundance table, using the log(x+1) cytokine responses as the response variable.

### Serology testing for common pathogens of mice

Serum samples obtained from two randomly selected mice per wedge in the outdoor enclosure were collected at the end of the experiment and sent to Charles River Research Animal Diagnostic Services to screen for antibodies against common pathogens infecting laboratory mice. Mice chosen for screening included those that displayed high levels of IFNγ or IL-17 in their LPMCs, which may be indicative of potential microbial infections. Serum samples were tested against sendai virus, pneumonia virus of mice, mouse hepatitis virus, minute virus of mice, mouse parvovirus 1 and 2, parvovirus NS-1, murine norovirus, theiler’s murine encephalomyelitis virus, reovirus, rat rotavirus, lymphocytic choriomeningitis virus, ectromelia virus (mousepox), adenovirus 1 and 2, mouse pneumonitis virus, polyoma virus, and *Mycoplasma pulmonis* (the Charles River Assessment Plus profile).

### Isolation and cytokine secretion profiles of LPMCs and MLNs

Isolation of LPMCs was carried out as previously described [83] (S1 Text) to measure gut mucosal cytokine responses from a randomized subset of mice. Subset numbers were imposed by daily processing limitations, and each time point (i.e., 3 weeks p.i. or 4 weeks p.i.) was represented by multiple sacrifice days. Sample sizes can be found in the appropriate figure legends. Isolated cells were stimulated together with 50 ng/mL PMA, 500 ng/mL Ionomycin, and brefeldin A for 4 hours at 37°C. Treatment with PMA and Ionomycin induces activation of cells to produce cytokines, and brefeldin A, a protein transport inhibitor, helps keep secreted cytokines within cells for intracellular cytokine staining. Following stimulation, cells were first stained extracellularly with a live/dead marker, anti-CD3, anti-CD4, and anti-CD8. After a fixation and permeabilization step, cells were stained with anti-IL-13, anti-IL-4, anti-IFNγ, anti-TNFα, anti-IL-10, and anti-IL-17A (S1 Text). Samples were acquired on an LSRII (Becton Dickinson [BD] Biosciences) and analyzed with the FlowJo (Tree Star, Ashland, OR) software. Cytokine-positive cells were quantified among single, live CD4+ and CD8+ T cells within the CD3+ population (S6A Fig).

Single cell suspensions were prepared from MLNs to measure intestinal cytokine responses to *T*. *muris* infection as previously described [84] (S1 Text). Concentrations of IL-13, IFNγ, IL-17, and IL-10 were determined in MLN culture supernatants using half-reactions of the Beckton Dickinson Cytometric Bead Array kit (BD Biosciences, UK; S1 Text). Samples were acquired on an LSRII (BD) and analyzed with the FCAP Array software (BD, Oxford, UK).

### Statistical analyses

All immunoparasitological data were analyzed using general or generalized linear models, as follows. Data on nematode biomass, cytokine-positive lamina propria cells, and in vitro cytokine secretion were all log_10_(x+1) transformed to meet assumptions of analysis, but no additional data required transformation. Nematode burden data were analyzed using a negative binomial error distribution; log-likelihood comparison confirmed the appropriateness of that distribution rather than Poisson. Data were analyzed separately for the Week 3 versus Week 4 experimental end points. Random effects of source cage (i.e., the group in which each mouse arrived from the vendor) and destination enclosure (i.e., the outdoor enclosure into which each mouse was released) were fitted in mixed models but explained negligible variance in all cases. To facilitate estimation of effect sizes for fixed predictors (as described below), final models excluded random effects. Data on the gut microbiota were analyzed as described above.

In models of nematode burdens and biomass for *T*. *muris*–inoculated C57BL/6 mice, the predictor variable of “location” (Lab, Short-term Wild, or Long-term Wild) was used, followed by the Tukey post hoc test for multiple comparisons. For the model of nematode burden in C57BL/6 versus STAT6-/- mice maintained for the short term outdoors, genotype was the predictor variable. In models of LPMC and MLN cytokine production, predictor variables of infection (nematode infected versus uninfected), location (as above), and their interaction were used, followed by the Tukey post hoc test for multiple comparisons. In models of body weight, predictor variables of infection, location, and their interaction (as above) were also used, along with a covariate of the initial weight of each individual. For depiction in figures, *P* values were categorized as significant according to the following levels: *P < 0.05, **P < 0.01, ***P < 0.001. Analyses were run in R version 3.1.2, using default glm functions plus the lme4 [85], MASS [86], and multcomp [87] packages.

Data deposited in the Dryad repository: http://dx.doi.org/10.5061/dryad.h9g697r [88].

## Supporting information

S1 Fig *Trichuris muris* worm burdens and worm biomass in C57BL/6 mice residing in laboratory versus outdoor environments at 4 weeks p.i. (**A**) Worm burdens and (**B**) worm biomass from infected C57BL/6 mice residing in laboratory and outdoor environments at 4 weeks p.i. Lab: *N* = 7, Short-term Wild: *N* = 7, Long-term Wild: *N* = 8. Box centers show the medians, and the upper and lower box edges correspond to the 25th and 75th percentiles. Whiskers extend 1.5 times the interquartile range. Asterisks denote significance as **P < 0.01. https://doi.org/10.5061/dryad.h9g697r. p.i., postinfection.
(TIF)

S2 Fig Effects of environmental variation and infection on host weight over time. Body weight in grams lost/gained over the course of the experiment from uninfected and infected mice residing in laboratory and outdoor environments. Data are means ± standard error mean. https://doi.org/10.5061/dryad.h9g697r.
(TIF)

S3 Fig Albumin, total protein, and leptin levels in blood between infected and uninfected mice across all environments at 3 weeks and 4 weeks p.i. (**A**) Plasma albumin, total protein, and leptin levels in uninfected and infected mice across all environments at 3 weeks p.i. Sample sizes: Uninfected Lab mice: *N* = 2; Infected Lab mice: *N* = 8; Uninfected Short-term Wild mice: *N* = 5; Infected Short-term Wild mice: *N* = 10; Uninfected Long-term Wild mice: *N* = 9; Infected Long-term Wild mice: *N* = 10. (**B**) Plasma albumin, total protein, and leptin levels in uninfected and infected mice across all environments at 4 weeks p.i. Sample sizes: Uninfected Lab mice: *N* = 3; Infected Lab mice: *N* = 7; Uninfected Short-term Wild mice: *N* = 5; Infected Short-term Wild mice: *N* = 7; Uninfected Long-term Wild mice: *N* = 7; Infected Long-term Wild mice: *N* = 8. Nutritional data were log(x+1) transformed to meet assumptions of analysis. Box centers show the medians, and the upper and lower box edges correspond to the 25th and 75th percentiles. Whiskers extend 1.5 times the interquartile range. https://doi.org/10.5061/dryad.h9g697r. p.i., postinfection.
(TIF)

S4 Fig Outdoor environments alter the microbial and fungal communities of the gut. (**A**) Taxa summary plots at the phylum level showing the microbiota composition of individual Lab mice (*N* = 47) compared to Long-term Wild (*N* = 28) mice, which had been residing outdoors for two weeks. The Lab group includes both Lab and Short-term Wild mice, as both groups were residing under laboratory conditions at this time (Week 0). (**B**) Bray-Curtis dissimilarity in the gut microbiota of fecal samples from uninfected and infected mice in laboratory and outdoor environments at Week 0 and Weeks 3 and 4 p.i. Separation of sampling time is depicted by 95% confidence ellipses. (**C**) Species richness in the gut microbiota of fecal samples from laboratory and outdoor environments before *T*. *muris* infection, as measured by Chao1. The Lab group includes both Lab and Short-term Wild mice, as both groups were residing under laboratory conditions at this time (Week 0). Box centers show the medians, and the upper and lower box edges correspond to the 25th and 75th percentiles. Whiskers extend 1.5 times the interquartile range. (**D**) Bacterial density, as measured by 16S gene copies/μg of DNA for fecal samples from laboratory and outdoor environments before *T*. *muris* infection. The Lab group includes both Lab and Short-term Wild mice, as both groups were residing under laboratory conditions at this time (Week 0). Asterisks denote significance as ***P < 0.001. Box centers show the medians, and the upper and lower box edges correspond to the 25th and 75th percentiles. Whiskers extend 1.5 times the interquartile range. (**E**) Taxa summary plots at the family level showing the fungal composition of uninfected and infected mice across all environments at Week 0 and at 3 weeks and 4 weeks p.i. Data represent the mean relative abundance. Blank spaces represent an absence of data for those groups. https://doi.org/10.5061/dryad.h9g697r. 16S rRNA gene sequences available at NCBI SRA: SRP132155. p.i., postinfection.
(TIF)

S5 Fig Alterations in the composition, diversity, and density of the fecal microbiota with high-dose *Trichuris muris* infection. (**A**) Log_2_ fold change of OTUs that differ between infected and uninfected mice residing in laboratory and outdoor environments at 4 weeks p.i. using DESeq2. Data shown have been filtered to include OTUs that have a log_2_ fold change >2 or <2 with a baseMean >20 to show the abundant OTUs that are most changed with infection. Bars are colored to direct the attention of readers to one main OTU, *Barnesiella*. Gray bars are used to show how *Barnesiella* is decreased in Lab mice but increased in Long-term Wild mice. Bars depict mean + standard error. Mice that had purged all worms were excluded from the infected group analyses. (**B**) Mean alpha diversity based on Shannon index of unfiltered microbiota data for fecal samples at 3 weeks and 4 weeks p.i. (**C**) Bacterial density as measured by 16S gene copies/μg of DNA for fecal samples at 3 weeks p.i. Box centers show the medians, and the upper and lower box edges correspond to the 25th and 75th percentiles. Whiskers extend 1.5 times the interquartile range. Statistical analyses were performed with a two-way ANOVA, followed by Tukey posttest for multiple comparisons using R. 16S rRNA gene sequences available at NCBI SRA: SRP132155. OTU; operational taxonomic unit; p.i., postinfection; uc, unclassified.
(TIF)

S6 Fig Differences in the relative abundance of LPMC and MLN cytokines in mice living in laboratory and outdoor environments at 3 weeks p.i. (**A**) Representative gating strategy for CD4+ and CD8+ cytokine analyses from LPMCs of a laboratory mouse. (**B**) Proportion of CD4+ cells that are producing IL-4, IL-10, IL-17, and TNFα in LPMCs from mice residing in laboratory and outdoor environments at 3 weeks p.i. Sample sizes: Uninfected Lab mice: *N* = 2; Infected Lab mice: *N* = 4; Uninfected Short-term Wild mice: *N* = 3; Infected Short-term Wild mice: *N* = 5; Uninfected Long-term Wild mice: *N* = 4; Infected Long-term Wild mice: *N* = 6. (**C**) Concentrations of IL-10 and IL-17 produced from MLNs of mice residing in laboratory and outdoor environments 3 weeks p.i. Sample sizes: Uninfected Lab mice: *N* = 2; Infected Lab mice: *N* = 8; Uninfected Short-term Wild mice: *N* = 5; Infected Short-term Wild mice: *N* = 10; Uninfected Long-term Wild mice: *N* = 9; Infected Long-term Wild mice: *N* = 10. (**D**) Proportion of CD8+ cells that are producing IFNγ and TNFα in LPMCs from mice residing in laboratory and outdoor environments at 3 weeks p.i. Sample sizes: Uninfected Lab mice: *N* = 2; Infected Lab mice: *N* = 4; Uninfected Short-term Wild mice: *N* = 3; Infected Short-term Wild mice: *N* = 5; Uninfected Long-term Wild mice: *N* = 4; Infected Long-term Wild mice: *N* = 6. Data on cytokine-positive lamina propria cells and in vitro cytokine secretion were log(x+1) transformed to meet assumptions of analysis. A two-way ANOVA was conducted to investigate the effects of location, infection, and a location by infection interaction effect on cytokine expression, followed by Tukey post hoc test for multiple comparisons. Box centers show the medians, and the upper and lower box edges correspond to the 25th and 75th percentiles. Whiskers extend 1.5 times the interquartile range. https://doi.org/10.5061/dryad.h9g697r. IFNγ, interferon-gamma; IL, interleukin; LPMC, lamina propria mononuclear cell; MLN, mesenteric lymph node; p.i., postinfection; TNFα, tumor necrosis factor-alpha.
(TIF)

S7 Fig Differences in the relative abundance of LPMC and MLN cytokines in mice living in laboratory and outdoor environments at 4 weeks p.i. (**A**) Proportion of CD4+ cells that are producing IL-13, IFNγ, IL-4, IL-10, IL-17, and TNFα in LPMCs of mice residing in laboratory and outdoor environments at 4 weeks p.i. Sample sizes: Uninfected Lab mice: *N* = 3; Infected Lab mice: *N* = 5; Uninfected Short-term Wild mice: *N* = 5; Infected Short-term Wild mice: *N* = 7; Uninfected Long-term Wild mice: *N* = 4; Infected Long-term Wild mice: *N* = 6. (**B**) Concentrations of IL-10 and IL-17 produced from MLNs of mice residing in laboratory and outdoor environments at 4 weeks p.i. Sample sizes: Uninfected Lab mice: *N* = 3; Infected Lab mice: *N* = 7; Uninfected Short-term Wild mice: *N* = 5; Infected Short-term Wild mice: *N* = 7; Uninfected Long-term Wild mice: *N* = 7; Infected Long-term Wild mice: *N* = 8. (**C**) Proportion of CD8+ cells that are producing IFNγ and TNFα in LPMCs from mice residing in laboratory and outdoor environments at 4 weeks p.i. Sample sizes: Uninfected Lab mice: *N* = 3; Infected Lab mice: *N* = 5; Uninfected Short-term Wild mice: *N* = 5; Infected Short-term Wild mice: *N* = 7; Uninfected Long-term Wild mice: *N* = 4; Infected Long-term Wild mice: *N* = 6. Data on cytokine-positive lamina propria cells and in vitro cytokine secretion were log(x+1) transformed to meet assumptions of analysis. A two-way ANOVA was conducted to investigate the effects of location, infection, and a location by infection interaction effect on LPMC cytokine expression, followed by Tukey post hoc test for multiple comparisons. Box centers show the medians, and the upper and lower box edges correspond to the 25th and 75th percentiles. Whiskers extend 1.5 times the interquartile range. https://doi.org/10.5061/dryad.h9g697r. IFNγ, interferon-gamma; IL, interleukin; LPMC, lamina propria mononuclear cell; MLN, mesenteric lymph node; p.i., postinfection; TNFα, tumor necrosis factor-alpha.
(TIF)

S8 Fig Cytokine production in LPMCs of infected C57BL/6 and STAT6-/- mice residing outdoors for the short term. Proportion of CD4+ cells that are producing IL-4, IL-10, IL-17, and TNFα from LPMCs of infected C57BL/6 (*N* = 4) and STAT6-/- (*N* = 5) mice residing outdoors for the short term. Data on cytokine-positive lamina propria cells were log(x+1) transformed to meet the assumptions of analysis. Box centers show the medians, and the upper and lower box edges correspond to the 25th and 75th percentiles. Whiskers extend 1.5 times the interquartile range. https://doi.org/10.5061/dryad.h9g697r. IL, interleukin; LPMC, lamina propria mononuclear cell; STAT6-/-, mouse deficient in STAT6; TNFα, tumor necrosis factor-alpha.
(TIF)

S1 Table Statistical table showing blood albumin, total protein, and leptin levels at 3 weeks and 4 weeks p.i. Two-way ANOVA results for plasma albumin (g/dl), total protein (g/dl), and leptin (pg/ml) levels at 3 weeks and 4 weeks p.i., with location and infection as main factors. Nutritional data were log(x+1) transformed to meet assumptions of analysis. When the interaction term is not significant, the main effect results come from a model from which the interaction is dropped. p.i., postinfection.
(XLSX)

S2 Table Statistical table showing the alpha diversity of 16S microbiota data for fecal samples collected at 3 weeks and 4 weeks p.i. Two-way ANOVA results showing the alpha diversity as represented by the Shannon index of microbiota data for fecal samples at 3 weeks and 4 weeks p.i. When the interaction term is not significant, the main effect results come from a model from which the interaction is dropped. p.i., postinfection.
(XLSX)

S3 Table Statistical table showing LPMC and MLN cytokine production at 3 weeks and 4 weeks p.i. Two-way ANOVA results for CD4+ and CD8+ LPMC and MLN cytokine production at 3 weeks and 4 weeks p.i. with location and infection as main factors. Data on cytokine-positive lamina propria cells and in vitro cytokine secretion were log(x+1) transformed to meet assumptions of analysis. When the interaction term is not significant, the main effect results come from a model from which the interaction is dropped. LPMC, lamina propria mononuclear cell; MLN, mesenteric lymph node; p.i., postinfection.
(XLSX)

S1 Text Supporting information. (DOC)

## Abbreviations

CXCL10: C-X-C motif chemokine ligand10
GxE: genotype by environment
IFNγ: interferon-gamma
IL: interleukin
IL-4R: IL-4 receptor
ITS: internal transcribed spacer
L1: larval stage 1
L2: larval stage 2
LPMC: lamina propria mononuclear cell
MLN: mesenteric lymph node
NYU: New York University
OTU: operational taxonomic unit
PERMANOVA: permutation-based multivariate ANOVA
p.i.: postinfection
RFID: radio-frequency identification
STAT6: signal transducer and activator of transcription 6
STAT6-/-: mouse deficient in STAT6
Th1: type 1
Th2: type 2
TNFα: tumor necrosis factor-alpha

## References

[1] Shaw DJ, Dobson AP. Patterns of macroparasite abundance and aggregation in wildlife populations: a quantitative review. Parasitology. 1995;111 Suppl:S111–27. . 8632918 10.1017/s0031182000075855

[2] Shaw DJ, Grenfell BT, Dobson AP. Patterns of macroparasite aggregation in wildlife host populations. Parasitology. 1998;117 (Pt 6):597–610. . 9881385 10.1017/s0031182098003448

[3] Lloyd-Smith JO, Schreiber SJ, Kopp PE, Getz WM. Superspreading and the effect of individual variation on disease emergence. Nature. 2005;438(7066):355–9. doi: 10.1038/nature04153 . 16292310 10.1038/nature04153 PMC7094981

[4] Lively CM. The effect of host genetic diversity on disease spread. Am Nat. 2010;175(6):E149–52. doi: 10.1086/652430 . 20388005 10.1086/652430

[5] Anderson RM, May RM. Infectious diseases of humans: dynamics and control. Oxford; New York: Oxford University Press; 1991 viii, 757 p. p.

[6] Quinnell RJ. Genetics of susceptibility to human helminth infection. Int J Parasitol. 2003;33(11):1219–31. . 13678637 10.1016/s0020-7519(03)00175-9

[7] Stear MJ, Boag B, Cattadori I, Murphy L. Genetic variation in resistance to mixed, predominantly Teladorsagia circumcincta nematode infections of sheep: from heritabilities to gene identification. Parasite Immunol. 2009;31(5):274–82. doi: 10.1111/j.1365-3024.2009.01105.x . 19388948 10.1111/j.1365-3024.2009.01105.x

[8] Mitchell SE, Rogers ES, Little TJ, Read AF. Host-parasite and genotype-by-environment interactions: temperature modifies potential for selection by a sterilizing pathogen. Evolution. 2005;59(1):70–80. . 15792228

[9] Theriot CM, Koenigsknecht MJ, Carlson PE Jr., Hatton GE, Nelson AM, Li B, et al Antibiotic-induced shifts in the mouse gut microbiome and metabolome increase susceptibility to Clostridium difficile infection. Nat Commun. 2014;5:3114 doi: 10.1038/ncomms4114 . 24445449 10.1038/ncomms4114 PMC3950275

[10] Via S. The evolution of phenotypic plasticity: what do we really know? In: Real LA, editor. Ecological genetics. Princeton, NJ: Princeton University Press; 1994 p. 35–57.

[11] Hunter DJ. Gene-environment interactions in human diseases. Nat Rev Genet. 2005;6(4):287–98. doi: 10.1038/nrg1578 . 15803198 10.1038/nrg1578

[12] Cucchi T, Auffray JC, Vigne JD. On the origin of the house mouse synanthropy and dispersal in the Near East and Europe: zooarchaeological review and perspectives In: Macholán M, Baird SJE, Munclinger P, Piálek J, editors. Evolution in Our Neighbourhood: The House Mouse as a Model in Evolutionary Research. Cambridge, U.K: Cambridge University Press; 2012 p. 65–93.

[13] Abolins S, King EC, Lazarou L, Weldon L, Hughes L, Drescher P, et al The comparative immunology of wild and laboratory mice, Mus musculus domesticus. Nat Commun. 2017;8:14811 doi: 10.1038/ncomms14811 . 28466840 10.1038/ncomms14811 PMC5418598

[14] Viney M, Lazarou L, Abolins S. The laboratory mouse and wild immunology. Parasite Immunol. 2015;37(5):267–73. doi: 10.1111/pim.12150 . 25303494 10.1111/pim.12150

[15] Rosshart SP, Vassallo BG, Angeletti D, Hutchinson DS, Morgan AP, Takeda K, et al Wild Mouse Gut Microbiota Promotes Host Fitness and Improves Disease Resistance. Cell. 2017;171(5):1015–28 e13. doi: 10.1016/j.cell.2017.09.016 . 29056339 10.1016/j.cell.2017.09.016 PMC6887100

[16] Villarino NF, LeCleir GR, Denny JE, Dearth SP, Harding CL, Sloan SS, et al Composition of the gut microbiota modulates the severity of malaria. Proc Natl Acad Sci U S A. 2016;113(8):2235–40. doi: 10.1073/pnas.1504887113 . 26858424 10.1073/pnas.1504887113 PMC4776451

[17] Beura LK, Hamilton SE, Bi K, Schenkel JM, Odumade OA, Casey KA, et al Normalizing the environment recapitulates adult human immune traits in laboratory mice. Nature. 2016;532(7600):512–6. doi: 10.1038/nature17655 . 27096360 10.1038/nature17655 PMC4871315

[18] Reese TA, Bi K, Kambal A, Filali-Mouhim A, Beura LK, Burger MC, et al Sequential Infection with Common Pathogens Promotes Human-like Immune Gene Expression and Altered Vaccine Response. Cell Host Microbe. 2016;19(5):713–9. doi: 10.1016/j.chom.2016.04.003 . 27107939 10.1016/j.chom.2016.04.003 PMC4896745

[19] Brooker S. Estimating the global distribution and disease burden of intestinal nematode infections: adding up the numbers—a review. Int J Parasitol. 2010;40(10):1137–44. doi: 10.1016/j.ijpara.2010.04.004 . 20430032 10.1016/j.ijpara.2010.04.004 PMC3034165

[20] Pullan RL, Smith JL, Jasrasaria R, Brooker SJ. Global numbers of infection and disease burden of soil transmitted helminth infections in 2010. Parasit Vectors. 2014;7(1):37 doi: 10.1186/1756-3305-7-37 . 24447578 10.1186/1756-3305-7-37 PMC3905661

[21] Scott ME. Heligmosomoides polygyrus (Nematoda): susceptible and resistant strains of mice are indistinguishable following natural infection. Parasitology. 1991;103 Pt 3:429–38. . 1780180 10.1017/s0031182000059953

[22] Scott ME. High transmission rates restore expression of genetically determined susceptibility of mice to nematode infections. Parasitology. 2006;132(Pt 5):669–79. doi: 10.1017/S0031182005009583 . 16393368 10.1017/S0031182005009583

[23] Lippens C, Guivier E, Faivre B, Sorci G. Reaction norms of host immunity, host fitness and parasite performance in a mouse—intestinal nematode interaction. Int J Parasitol. 2016;46(2):133–40. doi: 10.1016/j.ijpara.2015.10.003 . 26627846 10.1016/j.ijpara.2015.10.003

[24] Budischak SA, Hansen C, Caudron Q, Garnier R, Kartzinel TR, Pelczer I, et al Feeding immunity: physiological and behavioral responses to infection and resource limitation. Front Immunol. 2018; 8:1914 doi: 10.3389/fimmu.2017.01914 . 29358937 10.3389/fimmu.2017.01914 PMC5766659

[25] Gordon C.J. 1993 Temperature Regulation in Laboratory Rodents. Cambridge University Press, New York: 276 pages.

[26] Hayes KS, Bancroft AJ, Goldrick M, Portsmouth C, Roberts IS, Grencis RK. Exploitation of the intestinal microflora by the parasitic nematode Trichuris muris. Science. 2010;328(5984):1391–4. doi: 10.1126/science.1187703 . 20538949 10.1126/science.1187703 PMC3428897

[27] Vejzagic N, Adelfio R, Keiser J, Kringel H, Thamsborg SM, Kapel CM. Bacteria-induced egg hatching differs for Trichuris muris and Trichuris suis. Parasit Vectors. 2015;8:371 doi: 10.1186/s13071-015-0986-z . 26174801 10.1186/s13071-015-0986-z PMC4501204

[28] Cliffe LJ, Humphreys NE, Lane TE, Potten CS, Booth C, Grencis RK. Accelerated intestinal epithelial cell turnover: a new mechanism of parasite expulsion. Science. 2005;308(5727):1463–5. doi: 10.1126/science.1108661 . 15933199 10.1126/science.1108661

[29] Urban JF Jr., Noben-Trauth N, Donaldson DD, Madden KB, Morris SC, Collins M, et al IL-13, IL-4Ralpha, and Stat6 are required for the expulsion of the gastrointestinal nematode parasite Nippostrongylus brasiliensis. Immunity. 1998;8(2):255–64. . 9492006 10.1016/s1074-7613(00)80477-x

[30] Hou J, Schindler U, Henzel WJ, Ho TC, Brasseur M, McKnight SL. An interleukin-4-induced transcription factor: IL-4 Stat. Science. 1994;265(5179):1701–6. . 8085155 10.1126/science.8085155

[31] Else KJ, Finkelman FD, Maliszewski CR, Grencis RK. Cytokine-mediated regulation of chronic intestinal helminth infection. J Exp Med. 1994;179(1):347–51. . 8270879 10.1084/jem.179.1.347 PMC2191309

[32] Hu Y, Ellis BL, Yiu YY, Miller MM, Urban JF, Shi LZ, et al An extensive comparison of the effect of anthelmintic classes on diverse nematodes. PLoS ONE. 2013;8(7):e70702 doi: 10.1371/journal.pone.0070702 . 23869246 10.1371/journal.pone.0070702 PMC3712009

[33] Else KJ, Grencis RK. Cellular immune responses to the murine nematode parasite Trichuris muris. I. Differential cytokine production during acute or chronic infection. Immunology. 1991;72(4):508–13. . 1903765 PMC1384369

[34] Else KJ, Hultner L, Grencis RK. Cellular immune responses to the murine nematode parasite Trichuris muris. II. Differential induction of TH-cell subsets in resistant versus susceptible mice. Immunology. 1992;75(2):232–7. . 1532377 PMC1384699

[35] Levison SE, McLaughlin JT, Zeef LA, Fisher P, Grencis RK, Pennock JL. Colonic transcriptional profiling in resistance and susceptibility to trichuriasis: phenotyping a chronic colitis and lessons for iatrogenic helminthosis. Inflamm Bowel Dis. 2010;16(12):2065–79. doi: 10.1002/ibd.21326 . 20687192 10.1002/ibd.21326

[36] Schopf LR, Hoffmann KF, Cheever AW, Urban JF Jr., Wynn TA. IL-10 is critical for host resistance and survival during gastrointestinal helminth infection. J Immunol. 2002;168(5):2383–92. . 11859129 10.4049/jimmunol.168.5.2383

[37] Bancroft AJ, Else KJ, Grencis RK. Low-level infection with Trichuris muris significantly affects the polarization of the CD4 response. Eur J Immunol. 1994;24(12):3113–8. doi: 10.1002/eji.1830241230 . 7805740 10.1002/eji.1830241230

[38] Bancroft AJ, Else KJ, Humphreys NE, Grencis RK. The effect of challenge and trickle Trichuris muris infections on the polarisation of the immune response. Int J Parasitol. 2001;31(14):1627–37. . 11730790 10.1016/s0020-7519(01)00281-8

[39] Spoelstra K, Wikelski M, Daan S, Loudon AS, Hau M. Natural selection against a circadian clock gene mutation in mice. Proc Natl Acad Sci U S A. 2016;113(3):686–91. doi: 10.1073/pnas.1516442113 . 26715747 10.1073/pnas.1516442113 PMC4725470

[40] Hurst RJ, Else KJ. Trichuris muris research revisited: a journey through time. Parasitology. 2013;140(11):1325–39. doi: 10.1017/S0031182013001054 . 23965819 10.1017/S0031182013001054 PMC3761323

[41] Grencis RK. Immunity to helminths: resistance, regulation, and susceptibility to gastrointestinal nematodes. Annu Rev Immunol. 2015;33:201–25. doi: 10.1146/annurev-immunol-032713-120218 . 25533702 10.1146/annurev-immunol-032713-120218

[42] Klementowicz JE, Travis MA, Grencis RK. Trichuris muris: a model of gastrointestinal parasite infection. Semin Immunopathol. 2012;34(6):815–28. doi: 10.1007/s00281-012-0348-2 . 23053395 10.1007/s00281-012-0348-2 PMC3496546

[43] Chang J, Wescott RB. Infectivity, fecundity, and survival of Nematospiroides dubius in gnotobiotic mice. Exp Parasitol. 1972;32(3):327–34. . 4675136 10.1016/0014-4894(72)90060-4

[44] Wescott RB, Todd AC. A Comparison of the Development of Nippostrongylus Brasiliensis in Germ-Free and Conventional Mice. J Parasitol. 1964;50:138–43. . 14125156

[45] Wescott RB. Experimental Nematospiroides dubius infection in germfree and conventional mice. Exp Parasitol. 1968;22(2):245–9. . 5652501 10.1016/0014-4894(68)90099-4

[46] Przyjalkowski Z. Effect of intestinal flora and of a monoculture of E. coli on the development of intestinal and muscular Trichinella spiralis in mice. Bull Acad Pol Sci Biol. 1968;16(7):433–7. . 4884943

[47] Johnson J, Reid WM. Ascaridia galli (Nematoda): development and survival in gnotobiotic chickens. Exp Parasitol. 1973;33(1):95–9. . 4632462 10.1016/0014-4894(73)90013-1

[48] David LA, Maurice CF, Carmody RN, Gootenberg DB, Button JE, Wolfe BE, et al Diet rapidly and reproducibly alters the human gut microbiome. Nature. 2014;505(7484):559–63. doi: 10.1038/nature12820 . 24336217 10.1038/nature12820 PMC3957428

[49] Dea-Ayuela MA, Rama-Iniguez S, Bolas-Fernandez F. Enhanced susceptibility to Trichuris muris infection of B10Br mice treated with the probiotic Lactobacillus casei. Int Immunopharmacol. 2008;8(1):28–35. doi: 10.1016/j.intimp.2007.10.003 . 18068097 10.1016/j.intimp.2007.10.003

[50] Reynolds LA, Smith KA, Filbey KJ, Harcus Y, Hewitson JP, Redpath SA, et al Commensal-pathogen interactions in the intestinal tract: lactobacilli promote infection with, and are promoted by, helminth parasites. Gut Microbes. 2014;5(4):522–32. doi: 10.4161/gmic.32155 . 25144609 10.4161/gmic.32155 PMC4822684

[51] Holm JB, Sorobetea D, Kiilerich P, Ramayo-Caldas Y, Estelle J, Ma T, et al Chronic Trichuris muris Infection Decreases Diversity of the Intestinal Microbiota and Concomitantly Increases the Abundance of Lactobacilli. PLoS ONE. 2015;10(5):e0125495 doi: 10.1371/journal.pone.0125495 . 25942314 10.1371/journal.pone.0125495 PMC4420551

[52] Houlden A, Hayes KS, Bancroft AJ, Worthington JJ, Wang P, Grencis RK, et al Chronic Trichuris muris Infection in C57BL/6 Mice Causes Significant Changes in Host Microbiota and Metabolome: Effects Reversed by Pathogen Clearance. PLoS ONE. 2015;10(5):e0125945 doi: 10.1371/journal.pone.0125945 . 25938477 10.1371/journal.pone.0125945 PMC4418675

[53] Else KJ, Entwistle GM, Grencis RK. Correlations between worm burden and markers of Th1 and Th2 cell subset induction in an inbred strain of mouse infected with Trichuris muris. Parasite Immunol. 1993;15(10):595–600. . 7877836 10.1111/pim.1993.15.10.595

[54] Shoham S, Levitz SM. The immune response to fungal infections. Br J Haematol. 2005;129(5):569–82. doi: 10.1111/j.1365-2141.2005.05397.x . 15916679 10.1111/j.1365-2141.2005.05397.x

[55] Artis D, Potten CS, Else KJ, Finkelman FD, Grencis RK. Trichuris muris: host intestinal epithelial cell hyperproliferation during chronic infection is regulated by interferon-gamma. Exp Parasitol. 1999;92(2):144–53. doi: 10.1006/expr.1999.4407 . 10366539 10.1006/expr.1999.4407

[56] Dohms JE, Metz A. Stress—mechanisms of immunosuppression. Vet Immunol Immunopathol. 1991;30(1):89–109. . 1781159 10.1016/0165-2427(91)90011-z

[57] Antignano F, Mullaly SC, Burrows K, Zaph C. Trichuris muris infection: a model of type 2 immunity and inflammation in the gut. J Vis Exp. 2011;(51). doi: 10.3791/2774 . 21654621 10.3791/2774 PMC3415709

[58] Cliffe LJ, Grencis RK. The Trichuris muris system: a paradigm of resistance and susceptibility to intestinal nematode infection. Adv Parasitol. 2004;57:255–307. doi: 10.1016/S0065-308X(04)57004-5 . 15504540 10.1016/S0065-308X(04)57004-5

[59] Thomas MBB, S. Thermal biology in insect-parasite interactions. Trends Ecol Evol. 2003;18(7):344–50.

[60] Lazzaro BP, Flores HA, Lorigan JG, Yourth CP. Genotype-by-environment interactions and adaptation to local temperature affect immunity and fecundity in Drosophila melanogaster. PLoS Pathog. 2008;4(3):e1000025 doi: 10.1371/journal.ppat.1000025 . 18369474 10.1371/journal.ppat.1000025 PMC2265416

[61] Fels D, Kaltz O. Temperature-dependent transmission and latency of Holospora undulata, a micronucleus-specific parasite of the ciliate Paramecium caudatum. Proc Biol Sci. 2006;273(1589):1031–8. doi: 10.1098/rspb.2005.3404 . 16627290 10.1098/rspb.2005.3404 PMC1560244

[62] Alto BW, Lounibos LP, Mores CN, Reiskind MH. Larval competition alters susceptibility of adult Aedes mosquitoes to dengue infection. Proc Biol Sci. 2008;275(1633):463–71. doi: 10.1098/rspb.2007.1497 . 18077250 10.1098/rspb.2007.1497 PMC2289994

[63] Chase JM, Shulman RS. Wetland isolation facilitates larval mosquito density through the reduction of predators. Ecol Entomol. 2009;34(6).

[64] Orsted M, Schou MF, Kristensen TN. Biotic and abiotic factors investigated in two Drosophila species—evidence of both negative and positive effects of interactions on performance. Sci Rep. 2017;7:40132 doi: 10.1038/srep40132 . 28059144 10.1038/srep40132 PMC5216344

[65] Little TJ, Colegrave N. Caging and Uncaging Genetics. PLoS Biol. 2016;14(7):e1002525 doi: 10.1371/journal.pbio.1002525 . 27458971 10.1371/journal.pbio.1002525 PMC4961361

[66] Maizels RM, Nussey DH. Into the wild: digging at immunology's evolutionary roots. Nat Immunol. 2013;14(9):879–83. doi: 10.1038/ni.2643 . 23959175 10.1038/ni.2643

[67] Schindelin J, Arganda-Carreras I, Frise E, Kaynig V, Longair M, Pietzsch T, et al Fiji: an open-source platform for biological-image analysis. Nat Methods. 2012;9(7):676–82. doi: 10.1038/nmeth.2019 . 22743772 10.1038/nmeth.2019 PMC3855844

[68] Peters RH. The ecological implications of body size: Cambridge University Press; 1983.

[69] Lambden J, Johnson PT. Quantifying the biomass of parasites to understand their role in aquatic communities. Ecol Evol. 2013;3(7):2310–21. doi: 10.1002/ece3.635 . 23919172 10.1002/ece3.635 PMC3728967

[70] Kozich JJ, Westcott SL, Baxter NT, Highlander SK, Schloss PD. Development of a dual-index sequencing strategy and curation pipeline for analyzing amplicon sequence data on the MiSeq Illumina sequencing platform. Appl Environ Microbiol. 2013;79(17):5112–20. doi: 10.1128/AEM.01043-13 . 23793624 10.1128/AEM.01043-13 PMC3753973

[71] Schloss PD, Westcott SL, Ryabin T, Hall JR, Hartmann M, Hollister EB, et al Introducing mothur: open-source, platform-independent, community-supported software for describing and comparing microbial communities. Appl Environ Microbiol. 2009;75(23):7537–41. doi: 10.1128/AEM.01541-09 . 19801464 10.1128/AEM.01541-09 PMC2786419

[72] McMurdie PJ, Holmes S. phyloseq: an R package for reproducible interactive analysis and graphics of microbiome census data. PLoS ONE. 2013;8(4):e61217 doi: 10.1371/journal.pone.0061217 . 23630581 10.1371/journal.pone.0061217 PMC3632530

[73] Love MI, Huber W, Anders S. Moderated estimation of fold change and dispersion for RNA-seq data with DESeq2. Genome Biol. 2014;15(12):550 doi: 10.1186/s13059-014-0550-8 . 25516281 10.1186/s13059-014-0550-8 PMC4302049

[74] Oksanen JB, G.; Kindt R.; Legendre P; Minchin P.; O’Hara R.; Simpson G.; Solymos P.; Stevens M.; Wagner H. vegan: Community Ecology Package. R package version 2.3–5 2016.

[75] Wickham H. ggplot2: Elegant Graphics for Data Analysis: Springer-Verlag New York; 2009.

[76] McMurdie PJ, Holmes S. Waste not, want not: why rarefying microbiome data is inadmissible. PLoS Comput Biol. 2014;10(4):e1003531 doi: 10.1371/journal.pcbi.1003531 . 24699258 10.1371/journal.pcbi.1003531 PMC3974642

[77] Ishwaran H, Kogalur UB. Random Forests for Survival, Regression and Classification (RF-SRC), R package version 2.4.2 2017.

[78] Anderson MJ. A new method for non-parametric multivariate analysis of variance. Austral Ecol. 2001;26(1):32–46.

[79] Barman M, Unold D, Shifley K, Amir E, Hung K, Bos N, et al Enteric salmonellosis disrupts the microbial ecology of the murine gastrointestinal tract. Infect Immun. 2008;76(3):907–15. doi: 10.1128/IAI.01432-07 . 18160481 10.1128/IAI.01432-07 PMC2258829

[80] White TJ, Bruns T, Lee S, Taylor JW. Amplification and direct sequencing of fungal ribosomal RNA genes for phylogenetics In: Innis MA, Gelfand DH, Sninsky JJ, White TJ, editors. PCR Protocols: A Guide to Methods and Applications. New York: Academic Press; 1990 p. 315–22.

[81] Epp LS, Boessenkool S, Bellemain EP, Haile J, Esposito A, Riaz T, et al New environmental metabarcodes for analysing soil DNA: potential for studying past and present ecosystems. Mol Ecol. 2012;21(8):1821–33. doi: 10.1111/j.1365-294X.2012.05537.x . 22486821 10.1111/j.1365-294X.2012.05537.x

[82] Boyer F, Mercier C, Bonin A, Le Bras Y, Taberlet P, Coissac E. obitools: a unix-inspired software package for DNA metabarcoding. Mol Ecol Resour. 2016;16(1):176–82. doi: 10.1111/1755-0998.12428 . 25959493 10.1111/1755-0998.12428

[83] Cox LM, Yamanishi S, Sohn J, Alekseyenko AV, Leung JM, Cho I, et al Altering the intestinal microbiota during a critical developmental window has lasting metabolic consequences. Cell. 2014;158(4):705–21. doi: 10.1016/j.cell.2014.05.052 . 25126780 10.1016/j.cell.2014.05.052 PMC4134513

[84] Bowcutt R, Bell LV, Little M, Wilson J, Booth C, Murray PJ, et al Arginase-1-expressing macrophages are dispensable for resistance to infection with the gastrointestinal helminth Trichuris muris. Parasite Immunol. 2011;33(7):411–20. doi: 10.1111/j.1365-3024.2011.01300.x . 21585399 10.1111/j.1365-3024.2011.01300.x PMC3644868

[85] Bates DM, M.; Bolker B.; Walker S. Fitting Linear Mixed-Effects Models Using lme4. Journal of Statistical Software. 2015;67(1):1–48.

[86] Venables WN, Ripley BD, Venables WN. Modern applied statistics with S. 4th ed. New York: Springer; 2002 xi, 495 p. p.

[87] Hothorn T, Bretz F, Westfall P. Simultaneous inference in general parametric models. Biom J. 2008;50(3):346–63. doi: 10.1002/bimj.200810425 . 18481363 10.1002/bimj.200810425

[88] Leung J. M. (2018) Data from: Rapid environmental effects on gut nematode susceptibility in rewilded mice. Dryad Digital Repository Openly available via http://dx.doi.org/10.5061/dryad.h9g697r 10.1371/journal.pbio.2004108 PMC5843147 29518091

