# Supplementary figures and images for: Rapid environmental effects on gut nematode susceptibility in rewilded mice
Source: PLoS Biol. 2018 Mar 8;16(3):e2004108. doi: 10.1371/journal.pbio.2004108 (PMC5843147; doi:10.1371/journal.pbio.2004108)

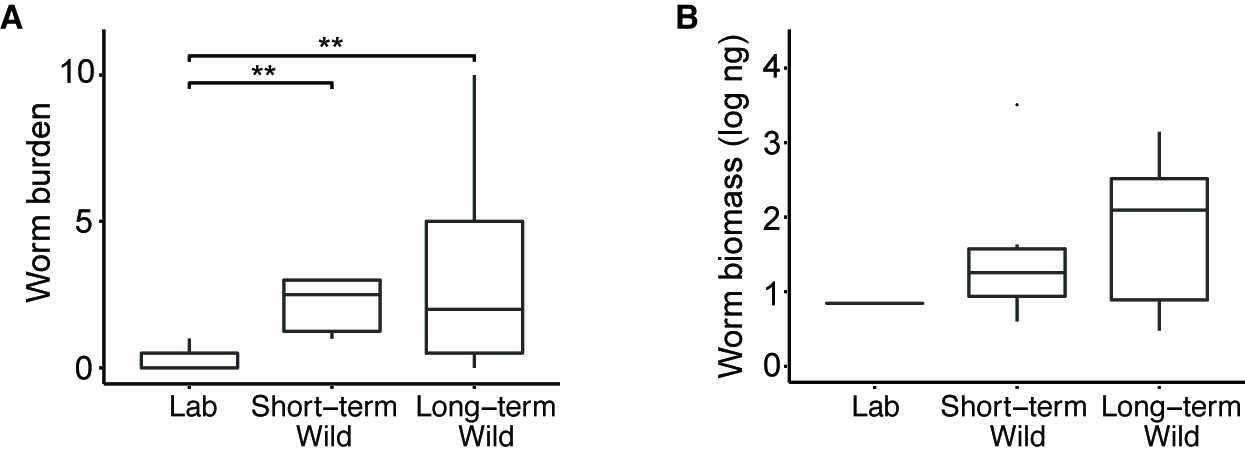

Supplement: S1 Fig — (A) Worm burdens and (B) worm biomass from infected C57BL/6 mice residing in laboratory and outdoor environments at 4 weeks p.i. Lab: N = 7, Short-term Wild: N = 7, Long-term Wild: N = 8. Box centers show the medians, and the upper and lower box edges correspond to the 25th and 75th percentiles. Whiskers extend 1.5 times the interquartile range. Asterisks denote significance as **P < 0.01. https://doi.org/10.5061/dryad.h9g697r. p.i., postinfection. (TIF) [file pbio.2004108.s001.tif]

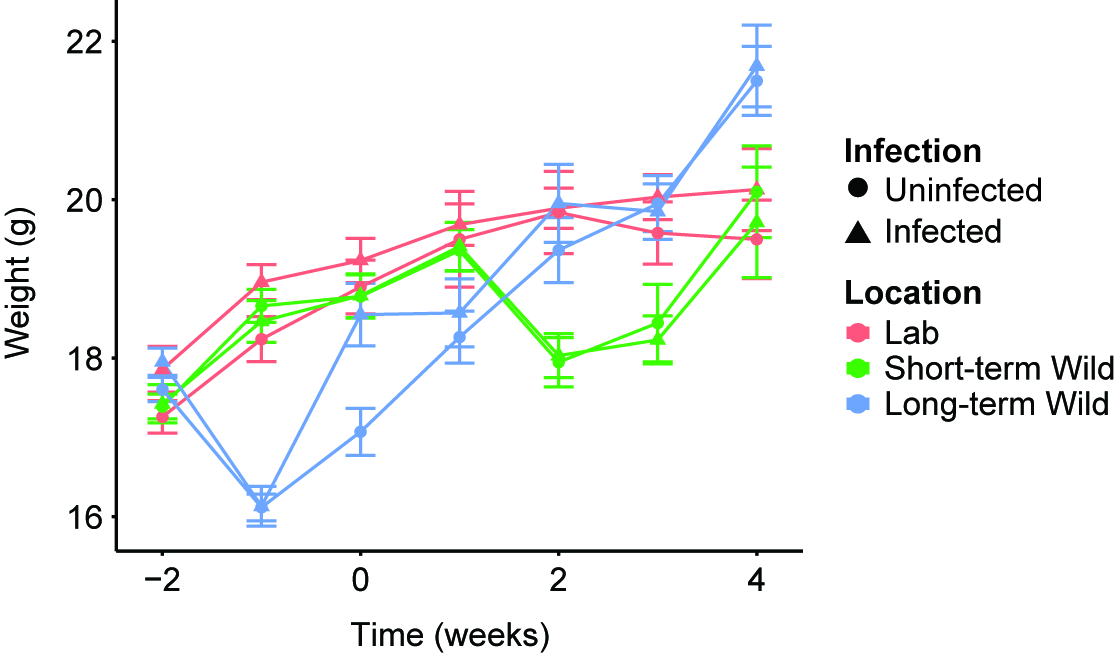

Supplement: S2 Fig — Body weight in grams lost/gained over the course of the experiment from uninfected and infected mice residing in laboratory and outdoor environments. Data are means ± standard error mean. https://doi.org/10.5061/dryad.h9g697r. (TIF) [file pbio.2004108.s002.tif]

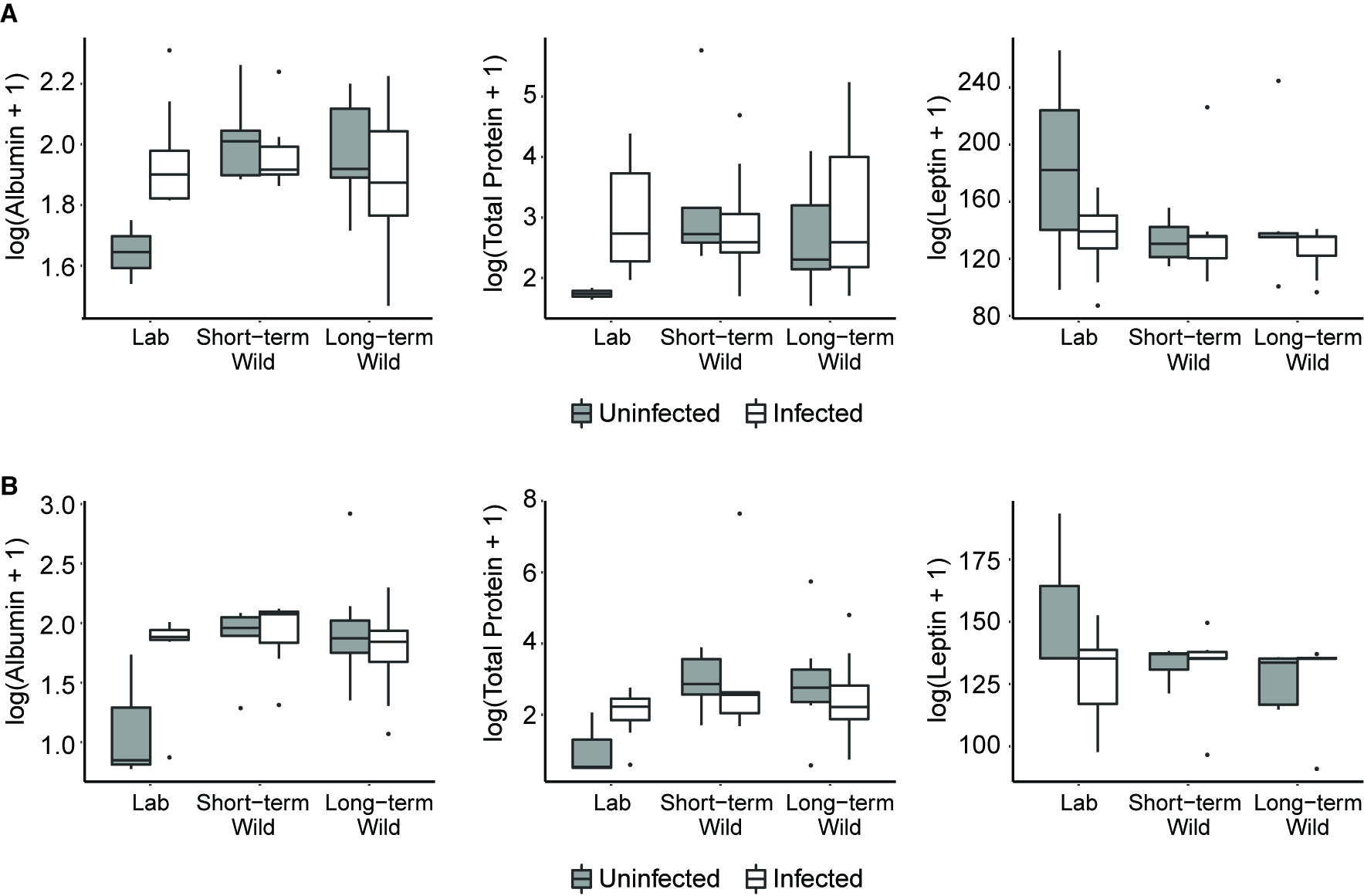

Supplement: S3 Fig — (A) Plasma albumin, total protein, and leptin levels in uninfected and infected mice across all environments at 3 weeks p.i. Sample sizes: Uninfected Lab mice: N = 2; Infected Lab mice: N = 8; Uninfected Short-term Wild mice: N = 5; Infected Short-term Wild mice: N = 10; Uninfected Long-term Wild mice: N = 9; Infected Long-term Wild mice: N = 10. (B) Plasma albumin, total protein, and leptin levels in uninfected and infected mice across all environments at 4 weeks p.i. Sample sizes: Uninfected Lab mice: N = 3; Infected Lab mice: N = 7; Uninfected Short-term Wild mice: N = 5; Infected Short-term Wild mice: N = 7; Uninfected Long-term Wild mice: N = 7; Infected Long-term Wild mice: N = 8. Nutritional data were log(x+1) transformed to meet assumptions of analysis. Box centers show the medians, and the upper and lower box edges correspond to the 25th and 75th percentiles. Whiskers extend 1.5 times the interquartile range. https://doi.org/10.5061/dryad.h9g697r. p.i., postinfection. (TIF) [file pbio.2004108.s003.tif]

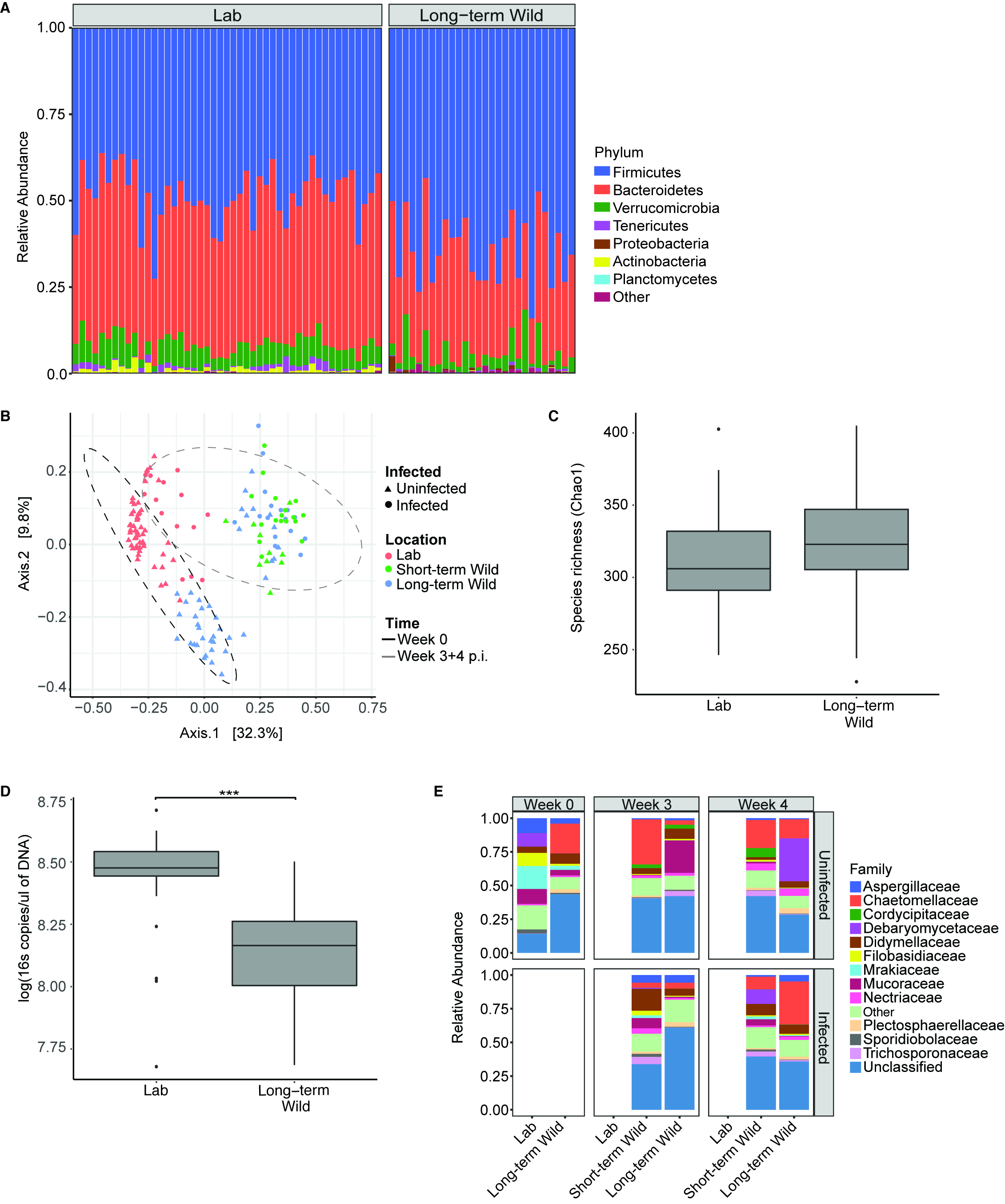

Supplement: S4 Fig — (A) Taxa summary plots at the phylum level showing the microbiota composition of individual Lab mice (N = 47) compared to Long-term Wild (N = 28) mice, which had been residing outdoors for two weeks. The Lab group includes both Lab and Short-term Wild mice, as both groups were residing under laboratory conditions at this time (Week 0). (B) Bray-Curtis dissimilarity in the gut microbiota of fecal samples from uninfected and infected mice in laboratory and outdoor environments at Week 0 and Weeks 3 and 4 p.i. Separation of sampling time is depicted by 95% confidence ellipses. (C) Species richness in the gut microbiota of fecal samples from laboratory and outdoor environments before T. muris infection, as measured by Chao1. The Lab group includes both Lab and Short-term Wild mice, as both groups were residing under laboratory conditions at this time (Week 0). Box centers show the medians, and the upper and lower box edges correspond to the 25th and 75th percentiles. Whiskers extend 1.5 times the interquartile range. (D) Bacterial density, as measured by 16S gene copies/μg of DNA for fecal samples from laboratory and outdoor environments before T. muris infection. The Lab group includes both Lab and Short-term Wild mice, as both groups were residing under laboratory conditions at this time (Week 0). Asterisks denote significance as ***P < 0.001. Box centers show the medians, and the upper and lower box edges correspond to the 25th and 75th percentiles. Whiskers extend 1.5 times the interquartile range. (E) Taxa summary plots at the family level showing the fungal composition of uninfected and infected mice across all environments at Week 0 and at 3 weeks and 4 weeks p.i. Data represent the mean relative abundance. Blank spaces represent an absence of data for those groups. https://doi.org/10.5061/dryad.h9g697r. 16S rRNA gene sequences available at NCBI SRA: SRP132155. p.i., postinfection. (TIF) [file pbio.2004108.s004.tif]

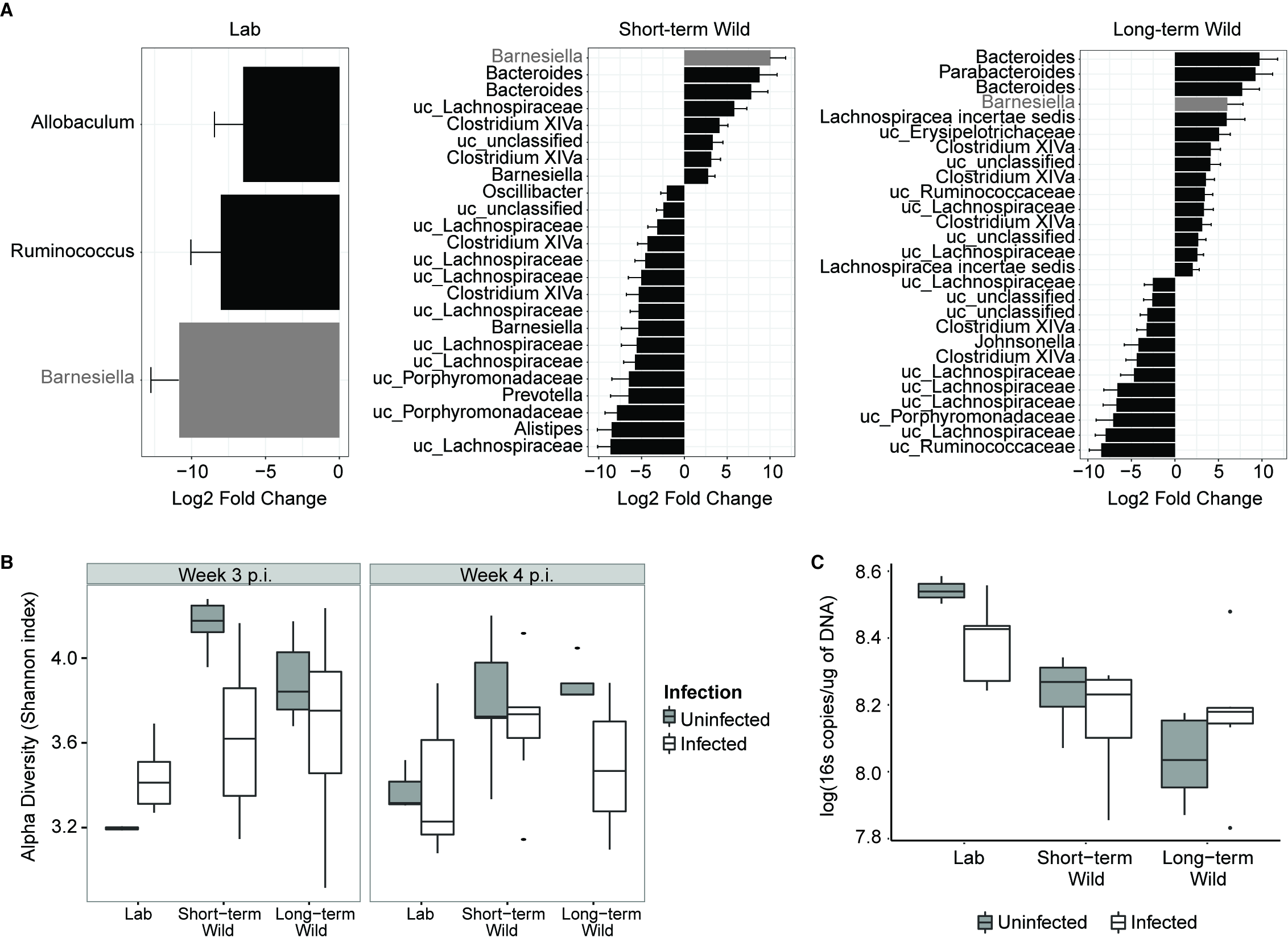

Supplement: S5 Fig — (A) Log2 fold change of OTUs that differ between infected and uninfected mice residing in laboratory and outdoor environments at 4 weeks p.i. using DESeq2. Data shown have been filtered to include OTUs that have a log2 fold change >2 or <2 with a baseMean >20 to show the abundant OTUs that are most changed with infection. Bars are colored to direct the attention of readers to one main OTU, Barnesiella. Gray bars are used to show how Barnesiella is decreased in Lab mice but increased in Long-term Wild mice. Bars depict mean + standard error. Mice that had purged all worms were excluded from the infected group analyses. (B) Mean alpha diversity based on Shannon index of unfiltered microbiota data for fecal samples at 3 weeks and 4 weeks p.i. (C) Bacterial density as measured by 16S gene copies/μg of DNA for fecal samples at 3 weeks p.i. Box centers show the medians, and the upper and lower box edges correspond to the 25th and 75th percentiles. Whiskers extend 1.5 times the interquartile range. Statistical analyses were performed with a two-way ANOVA, followed by Tukey posttest for multiple comparisons using R. 16S rRNA gene sequences available at NCBI SRA: SRP132155. OTU; operational taxonomic unit; p.i., postinfection; uc, unclassified. (TIF) [file pbio.2004108.s005.tif]

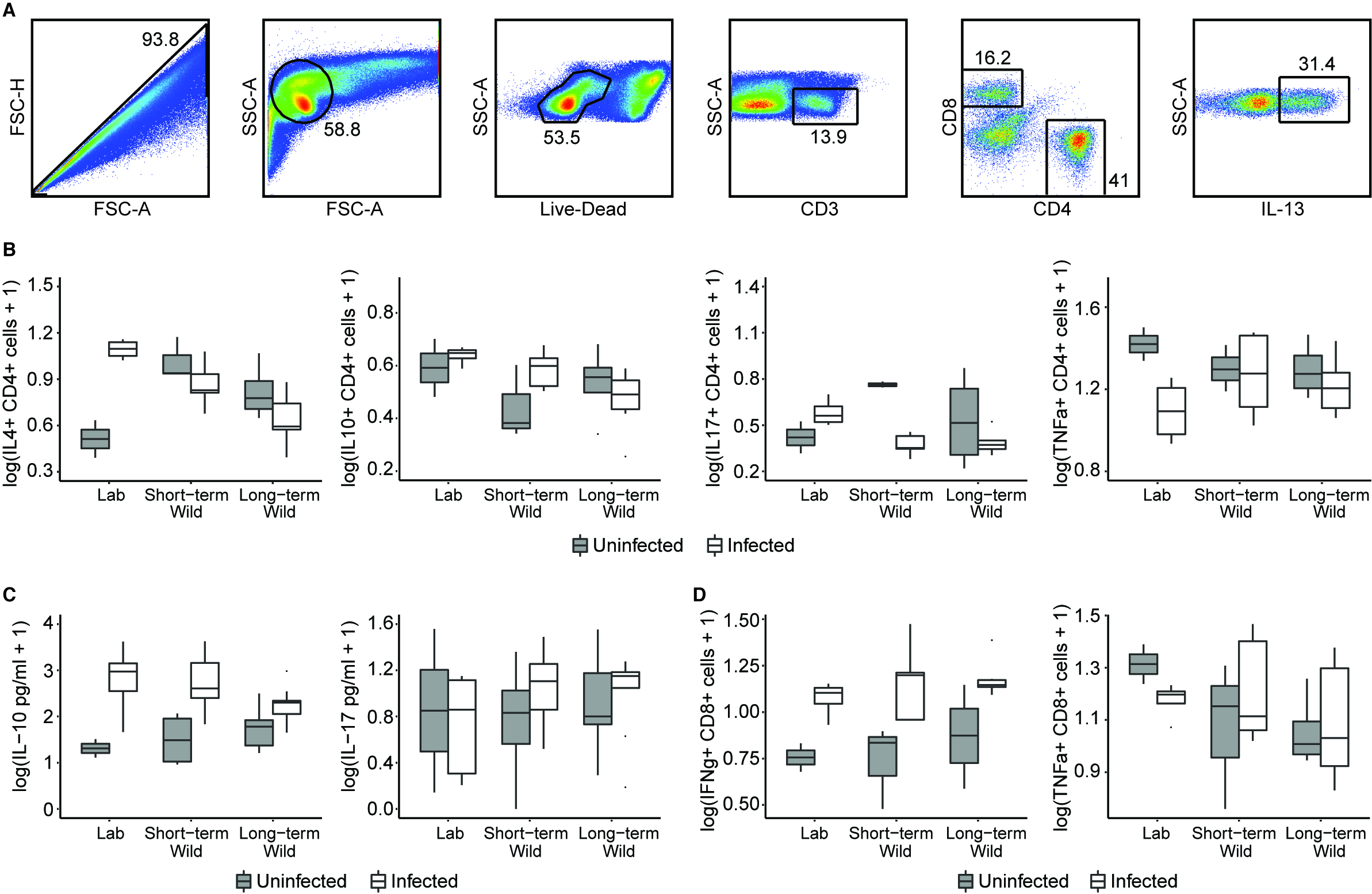

Supplement: S6 Fig — (A) Representative gating strategy for CD4+ and CD8+ cytokine analyses from LPMCs of a laboratory mouse. (B) Proportion of CD4+ cells that are producing IL-4, IL-10, IL-17, and TNFα in LPMCs from mice residing in laboratory and outdoor environments at 3 weeks p.i. Sample sizes: Uninfected Lab mice: N = 2; Infected Lab mice: N = 4; Uninfected Short-term Wild mice: N = 3; Infected Short-term Wild mice: N = 5; Uninfected Long-term Wild mice: N = 4; Infected Long-term Wild mice: N = 6. (C) Concentrations of IL-10 and IL-17 produced from MLNs of mice residing in laboratory and outdoor environments 3 weeks p.i. Sample sizes: Uninfected Lab mice: N = 2; Infected Lab mice: N = 8; Uninfected Short-term Wild mice: N = 5; Infected Short-term Wild mice: N = 10; Uninfected Long-term Wild mice: N = 9; Infected Long-term Wild mice: N = 10. (D) Proportion of CD8+ cells that are producing IFNγ and TNFα in LPMCs from mice residing in laboratory and outdoor environments at 3 weeks p.i. Sample sizes: Uninfected Lab mice: N = 2; Infected Lab mice: N = 4; Uninfected Short-term Wild mice: N = 3; Infected Short-term Wild mice: N = 5; Uninfected Long-term Wild mice: N = 4; Infected Long-term Wild mice: N = 6. Data on cytokine-positive lamina propria cells and in vitro cytokine secretion were log(x+1) transformed to meet assumptions of analysis. A two-way ANOVA was conducted to investigate the effects of location, infection, and a location by infection interaction effect on cytokine expression, followed by Tukey post hoc test for multiple comparisons. Box centers show the medians, and the upper and lower box edges correspond to the 25th and 75th percentiles. Whiskers extend 1.5 times the interquartile range. https://doi.org/10.5061/dryad.h9g697r. IFNγ, interferon-gamma; IL, interleukin; LPMC, lamina propria mononuclear cell; MLN, mesenteric lymph node; p.i., postinfection; TNFα, tumor necrosis factor-alpha. (TIF) [file pbio.2004108.s006.tif]

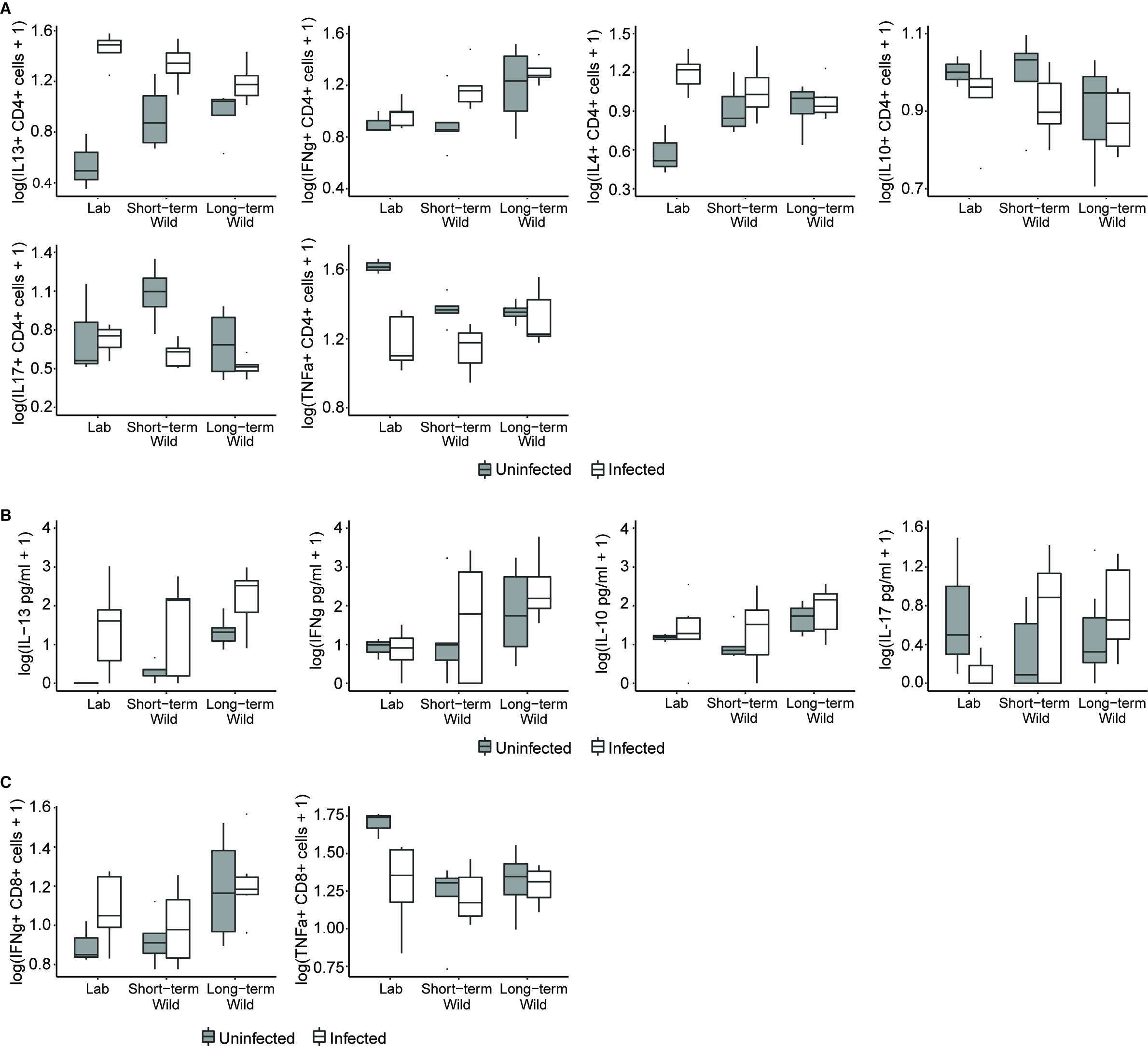

Supplement: S7 Fig — (A) Proportion of CD4+ cells that are producing IL-13, IFNγ, IL-4, IL-10, IL-17, and TNFα in LPMCs of mice residing in laboratory and outdoor environments at 4 weeks p.i. Sample sizes: Uninfected Lab mice: N = 3; Infected Lab mice: N = 5; Uninfected Short-term Wild mice: N = 5; Infected Short-term Wild mice: N = 7; Uninfected Long-term Wild mice: N = 4; Infected Long-term Wild mice: N = 6. (B) Concentrations of IL-10 and IL-17 produced from MLNs of mice residing in laboratory and outdoor environments at 4 weeks p.i. Sample sizes: Uninfected Lab mice: N = 3; Infected Lab mice: N = 7; Uninfected Short-term Wild mice: N = 5; Infected Short-term Wild mice: N = 7; Uninfected Long-term Wild mice: N = 7; Infected Long-term Wild mice: N = 8. (C) Proportion of CD8+ cells that are producing IFNγ and TNFα in LPMCs from mice residing in laboratory and outdoor environments at 4 weeks p.i. Sample sizes: Uninfected Lab mice: N = 3; Infected Lab mice: N = 5; Uninfected Short-term Wild mice: N = 5; Infected Short-term Wild mice: N = 7; Uninfected Long-term Wild mice: N = 4; Infected Long-term Wild mice: N = 6. Data on cytokine-positive lamina propria cells and in vitro cytokine secretion were log(x+1) transformed to meet assumptions of analysis. A two-way ANOVA was conducted to investigate the effects of location, infection, and a location by infection interaction effect on LPMC cytokine expression, followed by Tukey post hoc test for multiple comparisons. Box centers show the medians, and the upper and lower box edges correspond to the 25th and 75th percentiles. Whiskers extend 1.5 times the interquartile range. https://doi.org/10.5061/dryad.h9g697r. IFNγ, interferon-gamma; IL, interleukin; LPMC, lamina propria mononuclear cell; MLN, mesenteric lymph node; p.i., postinfection; TNFα, tumor necrosis factor-alpha. (TIF) [file pbio.2004108.s007.tif]

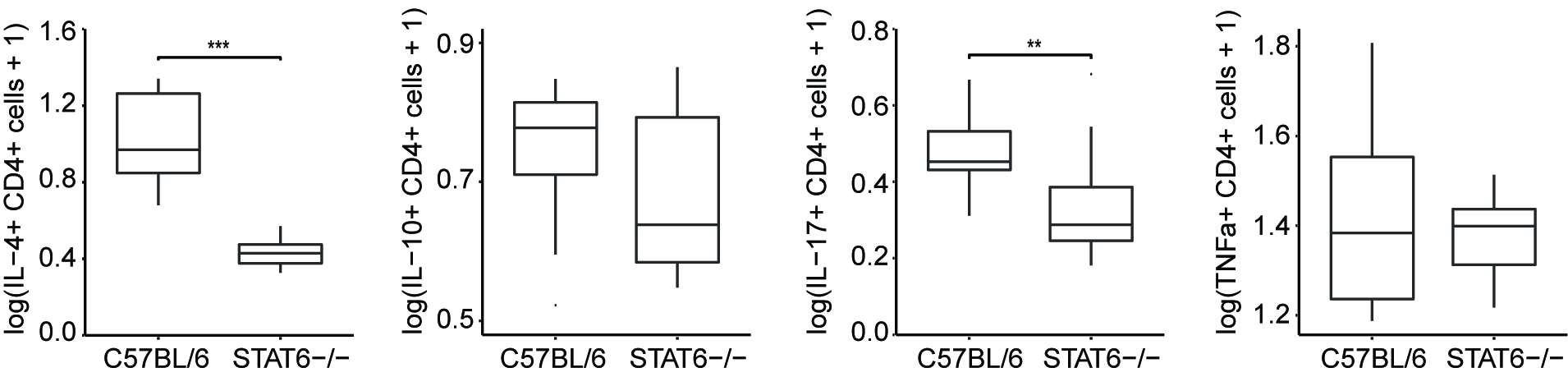

Supplement: S8 Fig — Proportion of CD4+ cells that are producing IL-4, IL-10, IL-17, and TNFα from LPMCs of infected C57BL/6 (N = 4) and STAT6-/- (N = 5) mice residing outdoors for the short term. Data on cytokine-positive lamina propria cells were log(x+1) transformed to meet the assumptions of analysis. Box centers show the medians, and the upper and lower box edges correspond to the 25th and 75th percentiles. Whiskers extend 1.5 times the interquartile range. https://doi.org/10.5061/dryad.h9g697r. IL, interleukin; LPMC, lamina propria mononuclear cell; STAT6-/-, mouse deficient in STAT6; TNFα, tumor necrosis factor-alpha. (TIF) [file pbio.2004108.s008.tif]
