# Supplementary material for: Rapid environmental effects on gut nematode susceptibility in rewilded mice
Source: PLoS Biol. 2018 Mar 8;16(3):e2004108. doi: 10.1371/journal.pbio.2004108 (PMC5843147; doi:10.1371/journal.pbio.2004108)
Supplement: S1 Text — (DOC) [file pbio.2004108.s012.doc]

**Rapid environmental effects on gut nematode susceptibility in rewilded mice**

Jacqueline M. Leung^1*^, Sarah A. Budischak^1^, Hao Chung The^2^, Christina Hansen^1^, Rowann Bowcutt^3^, Rebecca Neill^1^, Mitchell Shellman^1^, P’ng Loke^3^, Andrea L. Graham^1*^

**Supplementary Results and Discussion**

Other alterations to host physiology in C57BL/6 mice due to environmental change. Mice residing in the outdoor enclosures experienced many environmental changes compared to laboratory-maintained mice, including additional food resources, space availability, physical exercise, navigational challenges, and microbial exposure. It is thus unsurprising that traits other than gut community composition and immunological profiles differ between mice in the two locations. We did not attempt a comprehensive comparison of system-wide rapid effects of the changed environment. However, we did measure body weight as an overall index of condition (S2 Fig). All mice gained weight over the course of the experiment. We observed significant declines in weight during the week after each group moved outdoors, but weights rebounded within a few weeks (S2 Fig). By the end of the experiment, after controlling for initial weight (F(1,33)=5.61; P<0.0001), Long-term Wild mice weighed significantly more than Lab mice (|z|=2.91; P=0.0099) and trended towards weighing more than Short-term Wild mice (|z|=2.12; P=0.085). No significant differences in weight between helminth-infected and uninfected mice were observed, aside from a marginally non-significant interaction between location and infection in the week of *T. muris* inoculation (z=1.97; P=0.0542). This suggests that a coarse-grained index of nutritional plane does not correlate with nematode susceptibility changes.

Nonetheless, alterations in nutritional plane have been shown to alter helminth infection , and could thus play a role in *T. muris* susceptibility. Mice chow used in the lab was formulated with 20% protein (Picolab Rodent Diet 20). In *T. muris*-infected mice, a 4% protein deficiency has been shown to delay expulsion of adult worms . While we did provide the mice residing outdoors with unlimited supply of the same food chow used in the lab, these mice also had the ability to obtain food sources from within the wedges, such as berries, plants, and insects. No significant differences in blood leptin and total protein levels were observed between infected and uninfected mice across all environments at 3 weeks and 4 weeks p.i. (S3A Fig, S3B Fig, S1 Table). Future studies investigating the factors contributing to the weight changes we observed (e.g., in muscle or fat) will help disentangle how hosts are allocating their resources during nematode infection and may reveal that nutritional differences between lab and field help to explain the parasitological observations reported here.

Furthermore, movement of mice to the outdoor enclosures may lead to other changes that could potentially affect *T. muris* survival, including stress. The movement of mice to the outdoor enclosures may have induced stress in the mice, and this can be related to the dip in weight one week after hosts were moved outdoors. However, the weights of all mice rebounded quickly and reached similar weights to that of laboratory mice just one to two weeks later, suggesting that mice acclimated to the outdoor environments quite rapidly (S2 Fig).

Other cytokines measured in LPMCs and MLNs. We examined the relative proportions of CD4+ T cells producing IL-4, IL-10, IL-17, and TNFα in LPMCs of mice living in laboratory and outdoor environments at 3 weeks (S6B Fig, S3 Table) and 4 weeks (S7A Fig, S3 Table) p.i. At 3 weeks p.i., the proportion of CD4+ cells producing IL-4 followed the same trend as IL-13, with infected Long-term Wild mice harboring significantly higher proportions of CD4+ cells producing IL-4 than infected Lab mice. No significant differences in location, infection, or in the interaction between location and infection were observed in the proportion of CD4+ cells producing TNFα+ and IL-10+ cells. There was a significant main effect of infection and in the interaction between location and infection in the proportion of CD4+ cells producing IL-17. We also examined the relative abundance of IL10 and IL-17 in MLNs of mice residing in laboratory and outdoor environments at 3 weeks p.i. (S6C Fig). MLN concentrations of IL-10 showed a significant effect of location, infection, and the interaction between location and infection at 3 weeks p.i. For instance, both Lab and Short-term Wild infected mice had higher concentrations of IL-10 in MLNs than their uninfected counterparts. There were no significant differences in MLN IL-17 production among the different groups based on location and infection at this time point (S3 Table).

At 4 weeks p.i., infected Lab mice still exhibited the expected increase in IL-4 compared to uninfected Lab mice , as did Short-term Wild infected mice compared to their uninfected counterparts (S7A Fig, S3 Table). There were no significant differences in location, infection, or in the interaction between location and infection in the proportion of CD4+ cells producing IL-10 in LPCMs. There was a significant effect of infection, location, and in the interaction between infection and location in the proportion of CD4+ cells producing IL-17. There was also a significant effect of infection and in the interaction between infection and location on the proportion of TNFα-producing CD4+ cells (S7A Fig, S3 Table). In MLNs at 4 weeks p.i., there were no significant differences in the proportion of CD4+ cells producing IL-10 and IL-17 between uninfected and infected mice in the different location groups (S7B Fig, S3 Table).

Cytokines measured in C57BL/6 and STAT6-/- mice. The generation of type 2 cytokines, IL-4 and IL-13, is dependent upon the transcription factor, signal transducer and activator of transcription 6 (STAT6). As a result, mice deficient in STAT6 (STAT6-/-) are highly susceptible to nematode infections . Gating on single, live CD4+ T cells within the CD3+ population (S6A Fig), analysis of LPMCs from infected STAT6-/- mice revealed that the STAT6-/- mice were indeed deficient in the type 2 cytokines, IL-13 and IL-4 (Fig 4C, S8 Fig). Interestingly, the proportion of CD4+ cells producing IFNγ were indistinguishable between the two strains of infected mice when placed in outdoor enclosures (Fig 4C; P>0.05). There were no significant differences in the proportion of CD4+ cells producing IL-10 or TNFα between C57BL/6 and STAT6-/- mice residing outdoors for the short-term (S8 Fig). There was a significant difference in proportion of CD4+ cells producing IL-17 in LPMCs between these two genotypes (S8 Fig). These results indicate that residing in more natural environments skews essential nematode-clearing cytokines in C57BL/6 mice, which leads to worm burdens that become indistinguishable between STAT6-/- and C57BL/6 mice.

**Supplementary Materials and Methods**

Worm burden assessment. The cecum was removed at necropsy and frozen at -20°C until assessment of worm burdens, as follows. The cecum was thawed and cut longitudinally, and the contents were shaken out into a petri dish containing Millipore water. The cecal tissue was then moved into a clean petri dish with water, and the gut mucosa was scraped with forceps to release embedded worms. The tissue was then moved into a third petri dish and broken into smaller pieces in water. Adult and larval nematodes were counted in all three petri dishes under a dissection microscope, and the number of each life stage was noted. Worms were removed from each petri dish after counting to eliminate duplicate counts and placed in 100% ethanol for measurement of worm length and calculation of worm biomass.

16S rRNA sequencing and analyses. Sequences obtained from the Illumina MiSeq sequencer were analyzed using the mothur pipeline v 1.36.0 . Briefly, for each sample, the fastq files generated were first assembled into contigs and any sequences with ambiguous bases or a length longer than 275 bp were removed. Sequences from all samples were pooled for downstream analysis. Sequences were dereplicated and a count table was created to keep track of the number of unique sequences. Overhangs at both ends were removed so that only sequences aligning in the V4 region were obtained. Sequences with up to 2 nucleotide mismatches were grouped together, and chimeras were predicted and removed using UCHIME v 6.0, set at both *de novo* mode and against the ChimeraSlayer informed reference database. Sequences matching any Archaea, chloroplasts, and mitochondria sequences were removed. Singletons were removed from the dataset, and sequences were clustered into Operational Taxonomic Units (OTUs) at the level of Order and a cutoff of 0.03 (97% similarity), using the mothur’s clustering algorithm. Taxanomic assignments to each OTU up to the genus level were carried out using the mothur implemented Ribosomal Database Project (RDP) classifier, with at least 80% support in confidence. A cutoff of at least 10,000 sequences per sample was applied. Analyses in R were performed with an average of 61,458 ± 12,339 (SD) sequences per sample after filtering.

Determination of bacterial density in stool. DNA extracted from fecal samples collected at Week 0 and Week 3 was used for qPCR assessment of bacterial density using the 16s rRNA gene primers for Eubacteria, UniF340 (5-ACTCCTACGGGAGGCAGCAGT-3) and UniR514 (5-ATTACCGCGGCTGCTGGC-3) . Since we did not have stool weight and could not thus standardize our DNA output against grams of feces, we have used micrograms of DNA extracted as a normalization between samples. This may be an imperfect normalization because DNA samples from mice in different environments likely have varied DNA content from other sources (e.g., host DNA or DNA from supplementary feeding). Five fold dilutions of a plasmid standard constructed by cloning the 16s gene from Clostridia strains isolated from stool into a pcr2.1-TOPO plasmid was used for quantification of 16s gene copies. qPCR reactions were performed using the PowerUp SYBR Green Master Mix in a total reaction volume of 20μl. Reaction conditions consisted of an initial step of 50°C for 2 minutes followed by 95°C for 2 minutes. Forty cycles of 95°C for 15 seconds and 60°C for 60 seconds were then run, followed by conditions for a melting curve of 95°C for 15 seconds, 60°C for 60 seconds, and 95°C for 15 seconds to ensure that the qPCR reaction produced a single, specific product.

Isolation and flow cytometry of lamina propria mononuclear cells. Isolation of lamina propria mononuclear cells (LPMCs) was carried out as previously described . Briefly, the colons of a randomized subset of mice were removed at necropsy, cut longitudinally, and washed with HBSS. Epithelial cells were separated from the lamina propria through a 10 minute incubation in HBSS with HEPES, 5 mM EDTA, 1 mM DTT, and 1mM sodium pyruvate at 37°C with gentle shaking. Lamina propria tissue was pulse-vortexed and then incubated with HBSS, HEPES, 5mM EDTA, and 1mM sodium pyruvate at 37°C with gentle shaking for 15 minutes. The remaining tissue was washed in RPMI, finely chopped with scissors, and digested at 37°C for 20 minutes in a solution of RPMI with 1mg/ml Collagenase type IV, 40ug/ml DNaseI, 10% fetal bovine serum, and penicillin-streptomycin-glutamine. Cells were then forced through a 100-micron strainer and spun at 1500rpm for 5 minutes. LPMCs were isolated using a Percoll separation in which cells were resuspended in 40% percoll solution and overlaid on top of an 80% percoll solution. Cells were centrifuged without brake for 20 minutes at 2200rpm. LPMCs were collected at the interface, washed with HBSS, and used for subsequent flow cytometry analyses.

Cells were stimulated with 50 ng/ml PMA and 500 ng/ml Ionomycin for 4 hours at 37°C in the presence of brefeldin A. Following this *in vitro* stimulation, cells were stained with anti-CD3, anti-CD4, anti-CD8, and a live/dead stain and fixed in 4% paraformaldehyde in PBS. Cells were then permeabilized in Perm/Wash buffer (BD) and stained with anti-IL-13, anti-IL-4, anti-IFNγ, anti-TNFα, anti-IL-10, and anti-IL-17A. Cells were acquired on an LSRII (BD) and analyzed with FlowJo (Tree Star, Ashland, OR) software.

Isolation, culture, and cytokine secretion profiles of mesenteric lymph nodes. Mesenteric lymph nodes (MLN) were taken at autopsy and placed in sterile RPMI containing 10% fetal bovine serum and penicillin-streptomycin-glutamine. Single cell suspensions of MLN cells were prepared by forcing MLNs through a 100-micron cell strainer. Cells were counted on a hemocytometer and adjusted to a concentration of 5x10^6^ cells/ml. 500μl cultures in 48-well plates were stimulated with ‘4-hour’ *T. muris* E/S antigen at 5 μg/ml. Cells were incubated at 37°C, 5% CO_2_, 95% humidity for 48 hours, after which time supernatants were harvested and stored at –20°C until multiplex cytokine analysis by cytometric bead array.

Concentrations of IL-13, IFNγ, IL-17, and IL-10 were determined in MLN culture supernatants using half-reactions of the BD Cytometric Bead Array Mouse/Rat Soluble Protein Flex Set system (BD Biosciences, Oxford, UK). Briefly, lyophilised cytokine standards were pooled, reconstituted using assay diluent, and serial dilutions from 1:2 to 1:256 were prepared. The Protein Flex Set Capture Bead mix and Protein Flex Set Detection Reagent mix were prepared by diluting 1:50 with the capture bead diluent or detection bead diluent respectively. 0.5μl of each analyte bead was pooled and reconstituted in the total volume needed in capture bead or detection reagent diluent. 25 μl of capture bead mix and 25 μl of standard/sample was added to each tube and incubated for 1 hour. 25 μl of detection bead mixture was added to each tube, incubated for 1 hour, washed in wash buffer, and re-suspended in 150 μl of wash buffer. Cells were acquired on an LSRII (BD Biosciences) and analyzed with the FCAP Array software (BD Biosciences, Oxford, UK).

**Supplementary References**

Barman M, Unold D, Shifley K, Amir E, Hung K, Bos N, Salzman N. 2008. Enteric salmonellosis disrupts the microbial ecology of the murine gastrointestinal tract. *Infection and Immunity,* **76:**907-915. doi: 10.1128/IAI.01432-07

Cliffe LJ, Humphreys NE, Lane TE, Potten CS, Booth C, Grencis RK. 2005. Accelerated intestinal epithelial cell turnover: A new mechanism of parasite expulsion. *Science,* **308:**1463-1465. doi: 10.1126/science.1108661

Cox LM, Yamanishi S, Sohn J, Alekseyenko AV, Leung JM, Cho I, Kim SG, Li H, Gao Z, Mahana D, Zarate Rodriguez JG, Rogers AB, Robine N, Loke P, Blaser MJ. 2014. Altering the intestinal microbiota during a critical developmental window has lasting metabolic consequences. *Cell,* **158:**705-721. doi: 10.1016/j.cell.2014.05.052

Koski KG, Scott ME. 2001. Gastrointestinal nematodes, nutrition and immunity: Breaking the negative spiral. *Annual Review of Nutrition,* **21:**297-321. doi: 10.1146/annurev.nutr.21.1.297

Michael E, Bundy DA. 1991. The effect of the protein content of cba/ca mouse diet on the population dynamics of trichuris muris (nematoda) in primary infection. *Parasitology,* **103 Pt 3:**403-411.

Schloss PD, Westcott SL, Ryabin T, Hall JR, Hartmann M, Hollister EB, Lesniewski RA, Oakley BB, Parks DH, Robinson CJ, Sahl JW, Stres B, Thallinger GG, Van Horn DJ, Weber CF. 2009. Introducing mothur: Open-source, platform-independent, community-supported software for describing and comparing microbial communities. *Applied and Environmental Microbiology,* **75:**7537-7541. doi: 10.1128/AEM.01541-09

Urban JF, Jr., Noben-Trauth N, Donaldson DD, Madden KB, Morris SC, Collins M, Finkelman FD. 1998. Il-13, il-4ralpha, and stat6 are required for the expulsion of the gastrointestinal nematode parasite nippostrongylus brasiliensis. *Immunity,* **8:**255-264.
